# Co-expression of synaptic genes in the sponge *Amphimedon queenslandica* uncovers ancient neural submodules

**DOI:** 10.1038/s41598-019-51282-x

**Published:** 2019-10-31

**Authors:** Eunice Wong, Jan Mölter, Victor Anggono, Sandie M. Degnan, Bernard M. Degnan

**Affiliations:** 1 School of Biological Sciences, University of Queensland, Brisbane, Queensland 4072 Australia; 2 Queensland Brain Institute, University of Queensland, Brisbane, Queensland 4072 Australia; 3 School of Mathematics and Physics, University of Queensland, Brisbane, Queensland 4072 Australia; 4 Clem Jones Centre for Ageing Dementia Research, University of Queensland, Brisbane, Queensland 4072 Australia

**Keywords:** Evolutionary developmental biology, Gene expression

## Abstract

The synapse is a complex cellular module crucial to the functioning of neurons. It evolved largely through the exaptation of pre-existing smaller submodules, each of which are comprised of ancient sets of proteins that are conserved in modern animals and other eukaryotes. Although these ancient submodules themselves have non-neural roles, it has been hypothesized that they may mediate environmental sensing behaviors in aneural animals, such as sponges. Here we identify orthologues in the sponge *Amphimedon queenslandica* of genes encoding synaptic submodules in neural animals, and analyse their cell-type specific and developmental expression to determine their potential to be co-regulated. We find that genes comprising certain synaptic submodules, including those involved in vesicle trafficking, calcium-regulation and scaffolding of postsynaptic receptor clusters, are co-expressed in adult choanocytes and during metamorphosis. Although these submodules may contribute to sensory roles in this cell type and this life cycle stage, total synaptic gene co-expression profiles do not support the existence of a functional synapse in *A. queenslandica*. The lack of evidence for the co-regulation of genes necessary for pre- and post-synaptic functioning in *A. queenslandica* suggests that sponges, and perhaps the last common ancestor of sponges and other extant animals, had the ability to promulgate sensory inputs without complete synapse-like functionalities. The differential co-expression of multiple synaptic submodule genes in sponge choanocytes, which have sensory and feeding roles, however, is consistent with the metazoan ancestor minimally being able to undergo exo- and endocytosis in a controlled and localized manner.

## Introduction

Understanding the origin and the evolution of the nervous system and the neuron has remained an unresolved challenge despite research and debates spanning over a century^1–9^. Over the last decade, genomic and transcriptomic data, particularly from non-bilaterian metazoans (sponges, placozoans, cnidarians and ctenophores)^10–14^ and closely related unicellular holozoans (choanoflagellates, filastereans and ichthyosporeans)^15–17^ have shed light on the evolution of regulatory and structural gene families involved in neuron formation and function. However, the evolutionary gain and loss of neural features in early-branching metazoan phyla has been difficult to reconstruct^8,18–30^.

As with nested hierarchies in other biological systems^31–33^, one approach to reconstruct the origin of the neuron is to examine operational modules that contribute to its functionality, such as the synapse and its constitutive submodules^34,35^, in aneural (sponges and placozoans) and neural (ctenophores, cnidarians) non-bilaterian animals. Modules and their constituent submodules are composed of an assembly of biomolecules collectively performing a particular function. These collective performances are supported by the co-expression of gene products that participate in these common functions (e.g. signaling pathways and subcellular structures). As these gene products are often under the control of a shared transcriptional regulatory regime^32,36,37^, analysis and comparison of the expression of the genes comprising such modules provides a potential way to reconstruct the evolution of the neuron.

The origin of the synapse as an operational module of the neuron is critical to understanding the evolution of the nervous system^28,38,39^. Essential for building neural networks, the functional synapse is defined by a well-characterised set of co-regulated genes that can be assigned to specific synaptic submodules, including the post-synaptic density, synaptic vesicle and vacuolar-ATPase^36^. As is often the case, these and other synaptic submodules served other, often more ancient, biological functions prior to be being co-opted into the regulatory network underlying the functioning of the synapse^31,33,36,40^. These preexisting modules are able to retain their ancient functions as evolutionary selective pressure occurs primarily on the interactions between modules; internal connections within more ancient modules are typically more constrained and less evolvable^31,32,41^.

Most synaptic genes are present in non-bilaterian aneural animals and closely related unicellular holozoans^15,42–44^ and have been collectively termed the “protosynapse”^28,44–46^. However, there is a limited understanding of how these genes are expressed in these taxa^36,46,47^. Thus it has been difficult to gain insight into the regulatory relationship of synaptic submodules in aneural animals and how the synapse may have evolved. Here, we use a reassembled genome of the demosponge *Amphimedon queenslandica*, extensive developmental and cell type transcriptomes, and knowledge of cell type sensory functionality in larvae, juveniles and adults to infer the presence of synaptic submodules based on gene co-expression. Specifically, we target specific cell types and developmental stages with putative sensory functioning, including the adult choanocytes and pinacocytes that interface with the external environment, and the larval stage expressing neural genes^46,48,49^ and displaying phototactic behaviour^50^ and metamorphic cue detection^51^. This approach can allow insights into evolutionary and regulatory settings that may have shaped the evolution of the synapse and the neuron. In this process, we also compiled an updated list of orthologues of synaptic genes in *A. queenslandica*^46^.

## Results

### Synaptic genes in *Amphimedonqueenslandica*

**Figure 1:**
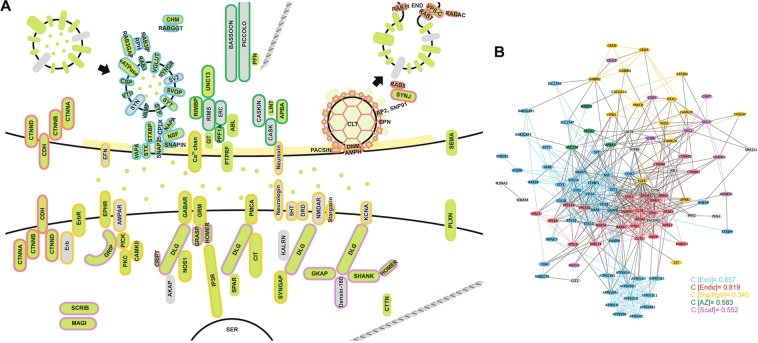
The synapse and its core submodules. (**A**) A diagram of the bilaterian synapse, signal transmitting and receiving cells, top and bottom respectively. Synaptic genes present in *Amphimedon queenslandica* are shaded green; genes not present are grey. Gene products are clustered into synaptic functions (submodules) and outlined by colour: exocytosis, blue; endocytosis, red; cell surface signals and receptors, yellow; active zone, dark green; and post-synaptic scaffolding, purple; gene products not outlined do not comprise these five submodules. (**B**) Evidence-based interactome for the human synaptome based on a non-redundant merging of BioGrid, STRING and APID databases. Genes (nodes) and associated interactions (edges/connecting lines) falling under the five core synaptic submodules are coded with the same color scheme as in (**A**). Clustering coefficients (**C**) for these submodules are shown.

Based on sequence similarity, phylogenetics, domain architecture and the presence of conserved motifs, we determined that *Amphimedon queenslandica* has 125 genes that are orthologues of the canonical human synaptome (Supplementary Fig. 1). These *A. queenslandica* genes provide a near-complete coverage of a functional synapse and largely can be categorised into one of the five functional synaptic groups: exocytosis; endocytosis; signaling/receptor system; active zone; and post-synaptic scaffolding (Fig. 1A). Synaptic gene orthologues apparently absent from the *A. queenslandica* genome include genes encoding trans-synaptic adhesion molecules neurexin and neuroligin, NMDA and AMPA ionotropic glutamate receptors, presynaptic adaptor CASK, vesicle surface protein synapsin and vesicle priming protein RIMS. Genes encoding serotonin and dopamine receptors, as well as voltage-gated potassium channels, are also absent from the *A. queenslandica* genome. The final merged synaptome generated from protein interaction databases BioGrid, STRING and APID consists of a total of 108 gene products (15 of the 125 *Amphimedon* synaptic genes are paralogues and two have no documented non-self interactions) connected by 691 interactions (Fig. 1B).

### Cell type and developmental co-expression of synaptic genes

**Figure 2:**
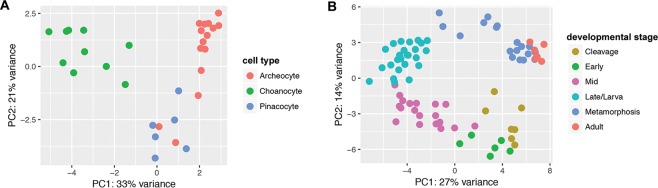
PCA of synaptic gene expression in *Amphimedon queenslandica*. Transcript counts cluster by (**A**) adult cell types and by (**B**) developmental stage; see Table 1 for descriptions of these.

Using CEL-Seq2, we assessed the expression of *Amphimedon* synaptic genes between stages of the sponge life cycle – embryonic, larval, postlarval, juvenile and adult^52^ and three types of manually isolated adult cells^53^. Principle component analysis (PCA) shows that the expression profiles of these synaptic genes cluster according to both developmental stage and cell type (Fig. 2).

**Figure 3:**
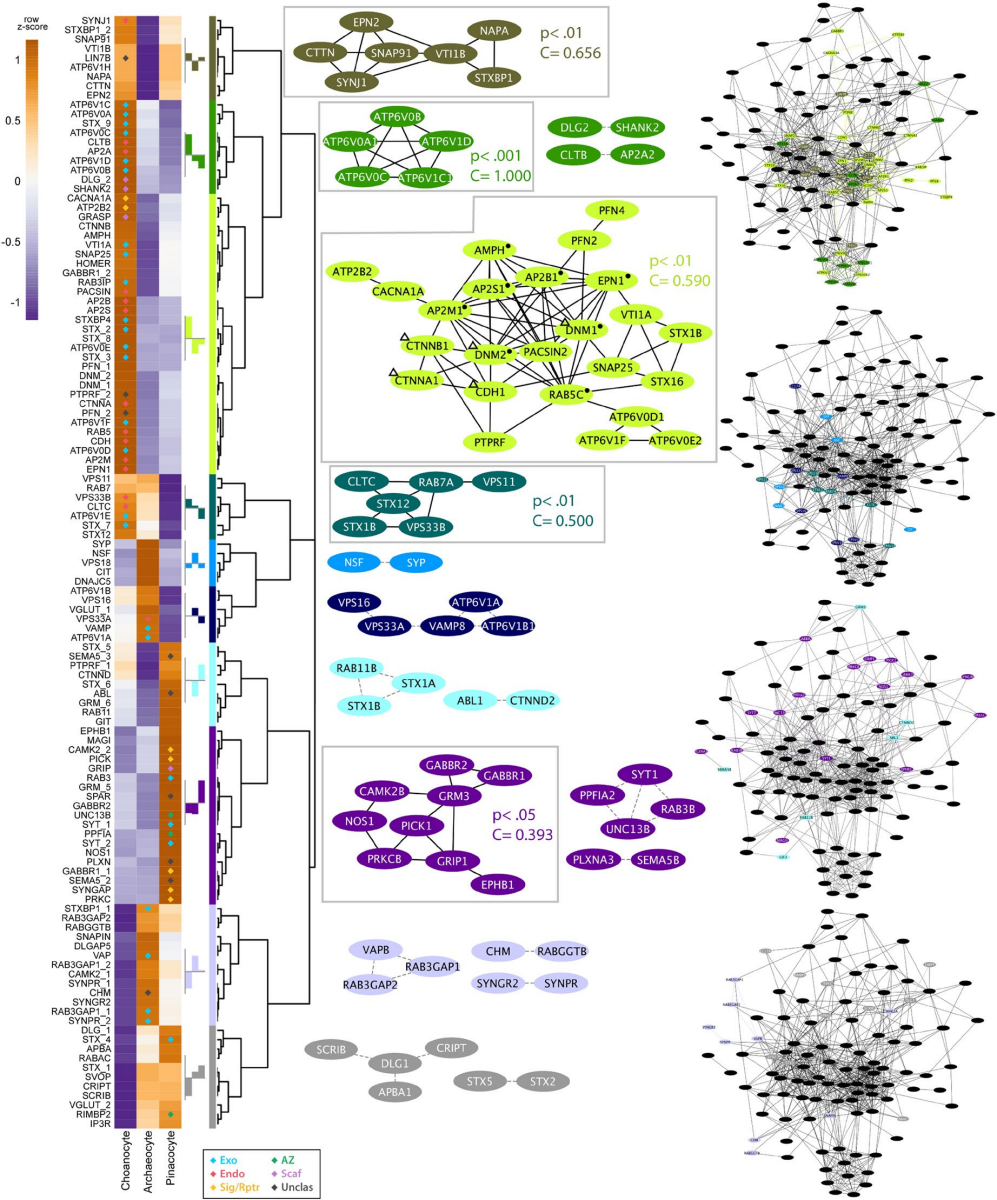
Cell type expression of synaptic genes in *Amphimedon queenslandica*. Heatmap to the left shows synaptic gene expression profiles across three adult cell types, with diamonds indicating statistically significant (p < 0.05) gene upregulation in corresponding cell type; colour-coding of the diamonds is in relation to synaptic function as per Fig. 1 – blue, exocytosis; red, endocytosis; yellow, cell surface signals and receptors; dark green, active zone; purple, post-synaptic scaffolding. Z-scores reflect expression levels (variance stablising transformed (vst) counts), scaled by rows. Genes are divided into ten clades (colour-codes) based on expression profile similarities. The consensus expression profile for each clade is shown to the left of the colour bar. For each clade, all inferred interactions are shown based on the human synaptome in Fig. 1B. Non-interacting nodes are not shown. Significant co-regulating modules supported by Monte Carlo analysis have black edges and are are shown with corresponding p-values and clustering coefficients (C); edges are otherwise grey dashed. To the right are the complete human synaptic interactome decorated with the genes from the four major clades (i.e. genes with co-localised expressions) grouped in the same synaptome. Symbols in the largest clade (lime) indicate genes mapped to the enriched pathways of *Endocytosis* (●) and *Bacterial invasion of epithelial cells* (Δ). See Supplementary Table 1 for a complete list of mapped pathways.

The majority of *Amphimedon* synaptic genes are upregulated during metamorphosis and in the adult, with the most substantial increase in expression of synaptic genes (37.6%) being when metamorphosis commences. Given this, we first focused on the expression of synaptic genes in adult cell types. We targeted three cell types that are essential for the sponge body plan: (i) choanocytes, internal epithelial feeding cells that form chambers that pump water through the sponge and capture exogenous microbes^54,55^; (ii) pinacocytes, epithelial cells that line internal canals and the outside of the sponge; and (iii) archeocytes, pluripotent stem cells that inhabit the middle collagenous mesohyl layer^56,57^. Both choanocytes and pinacocytes first appear during metamorphosis and directly interact with the external environment; archeocytes are present from embryogenesis onwards. The number of synaptic genes that are significantly upregulated in choanocytes (38; 30.4%) is more than double that of either pinacocytes (18; 14.4%) or archeocytes (8; 6.4%) (Fig. 3).

Based on cell type specific co-expression profiles, *Amphimedon* synaptic genes can be divided into ten clades, from which interactive network modules were generated (Fig. 3). Of these, five co-expression network modules were deemed significant. Four of these correspond to networks of genes that are highly expressed in choanocytes, most of which are significantly upregulated (olive, green, lime and teal modules, Fig. 3); the other significant co-expression network (purple) corresponds to genes upregulated in pinacocytes. In both cell types, the genes comprising the significant networks encode proteins that are part of multiple synaptic submodules (Fig. 3).

**Figure 4:**
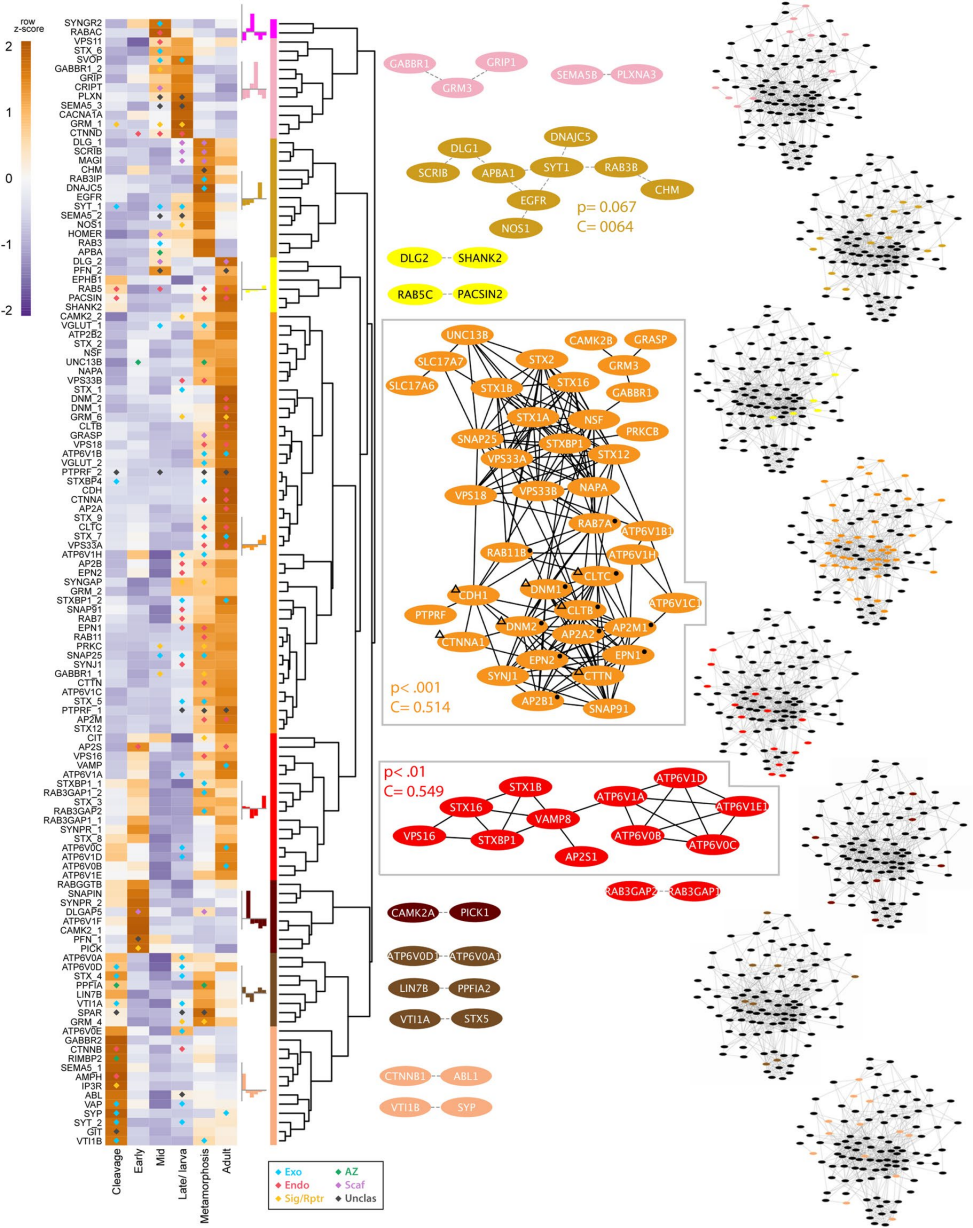
Developmental expression of synaptic genes in *Amphimedon queenslandica*. Heatmap shows synaptic gene expression profiles over six developmental stages, with diamonds indicating statistically significant (p < 0.05) gene upregulation compared to the previous stage (except for cleavage stage, where upregulation is with respect to the early embryogenesis stage); colour-coding is in relation to synaptic function as per Fig. 1 - blue, exocytosis; red, endocytosis; yellow, cell surface signals and receptors; dark green, active zone; purple, post-synaptic scaffolding. The generation of dendrogram, colour modules, mapped pathways and clustering coefficients are as described in Fig. 3. Symbols in the largest clade (orange) indicate genes mapped to the enriched pathways of *Endocytosis* (●) and *Bacterial invasion of epithelial cells* (Δ). See Supplementary Table 1 for a complete list of mapped pathways.

More synaptic genes are significantly upregulated in late developmental stages (late embryogenesis/larva, 46; metamorphosis, 47; adult, 32) than in early developmental stages (cleavage, 25; early embryogenesis, 9; mid-embryogenesis, 25) (Fig. 4). Based on developmental co-expression profile, genes were divided into eight clades (Fig. 4). Interactive network modules were generated for all but one clade (magenta), which consists of non-interacting genes. Two co-expression networks were significant and consisted of genes that are co-expressed at high levels during metamorphosis and in adults (orange and red modules, Fig. 4). These co-expressed genes comprise primarily exocytosis and endocytosis modules, and are highly expressed in choanocytes or pinacocytes (Fig. 3).

All cell type and developmental co-expression clades mapped to multiple biological pathways (Supplementary Table 1).

### Co-expression of synaptic submodule genes

#### Endocytosis

**Figure 5:**
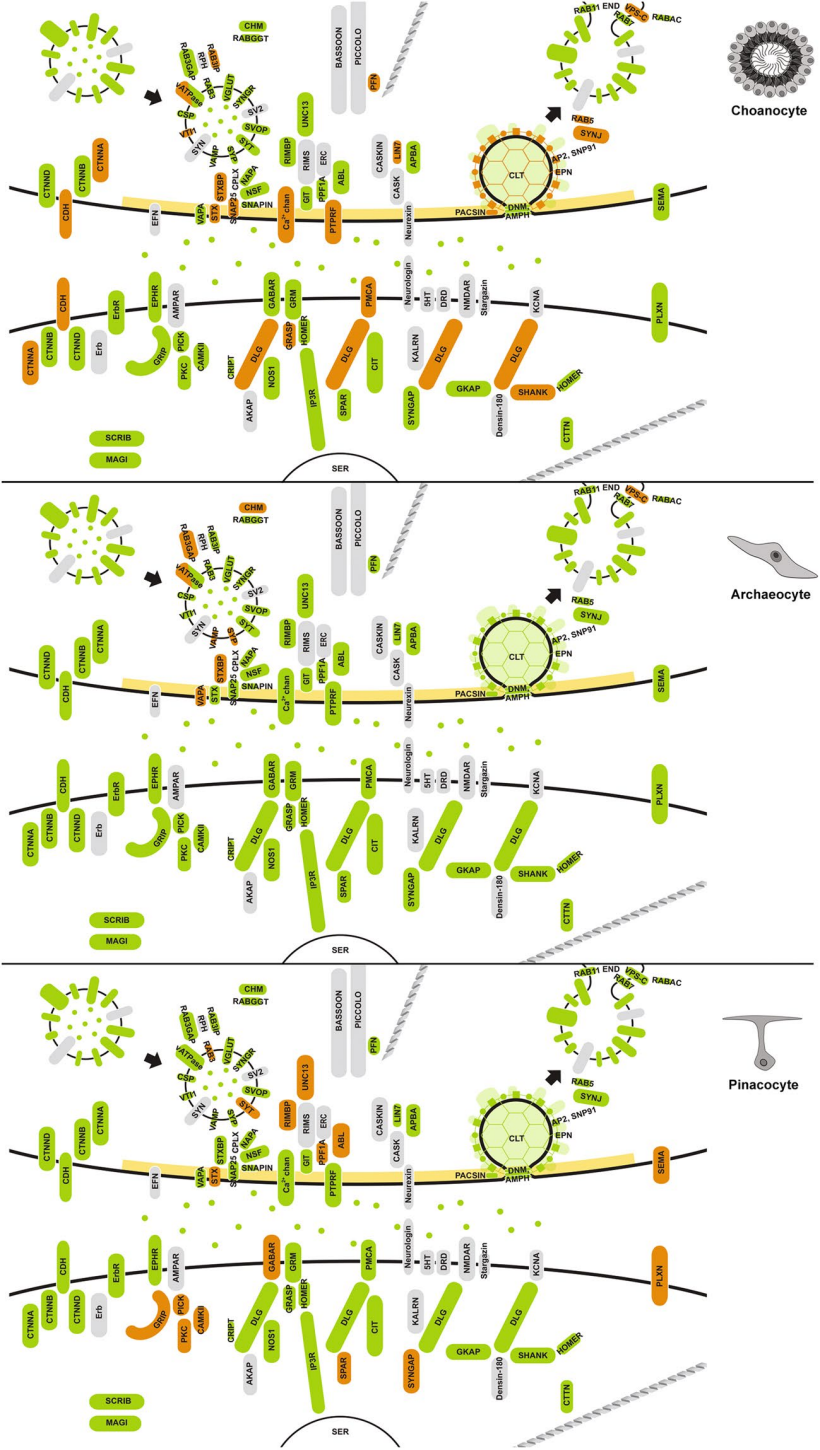
Upregulated synaptic genes in *Amphimedon queenslandica* cell types. Significantly upregulated synaptic genes (p-adj < 0.05) in each cell type, based on pairwise comparisons, are in orange. Green, orthologues of synaptic genes in *Amphimedon*; grey, genes not present in *Amphimedon*; yellow, active zone. Genes coding the endocytosis pathway (e.g. CLT, EPN, PACSIN), scaffolding and adhesion (e.g. DLG, GRASP, CDH) are enriched in choanocytes, while signaling/receptor genes (e.g. GABAR, CAMKII, SYNGAP) are upregulated in pinacocytes.

**Figure 6:**
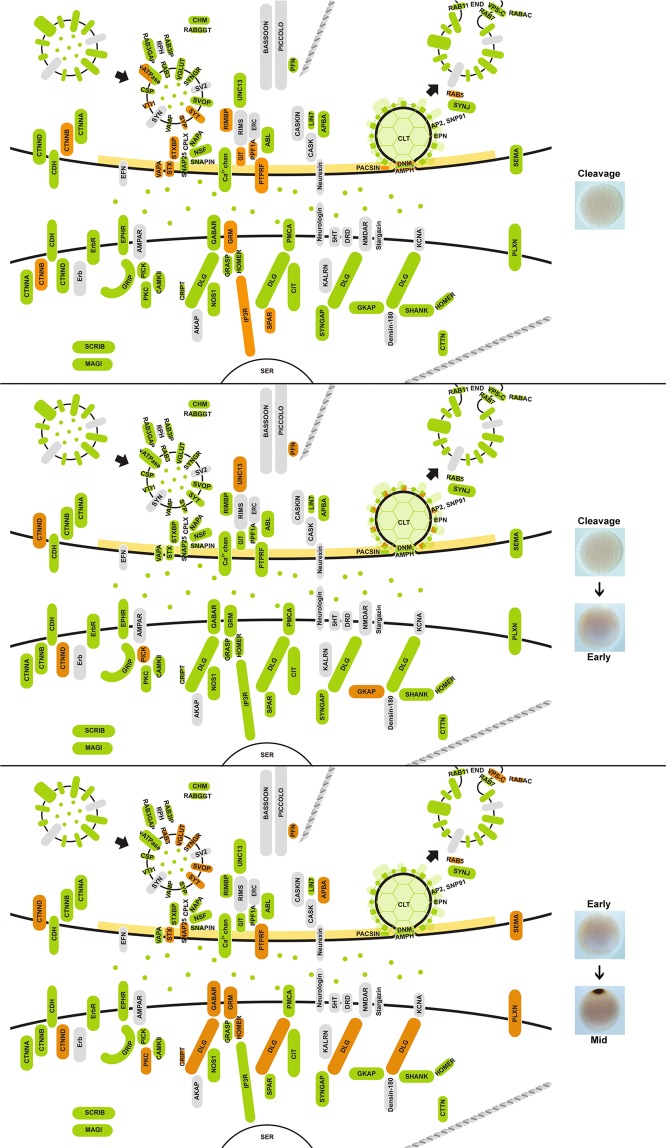
Upregulated synaptic genes during *Amphimedon queenslandica* embryogenesis. Significantly upregulated genes were identified by pairwise comparisons between life stages (p-adj < 0.05) and are coloured orange. Green, orthologues of synaptic genes in *Amphimedon*; grey, genes not present in *Amphimedon*; yellow, active zone.

**Figure 7:**
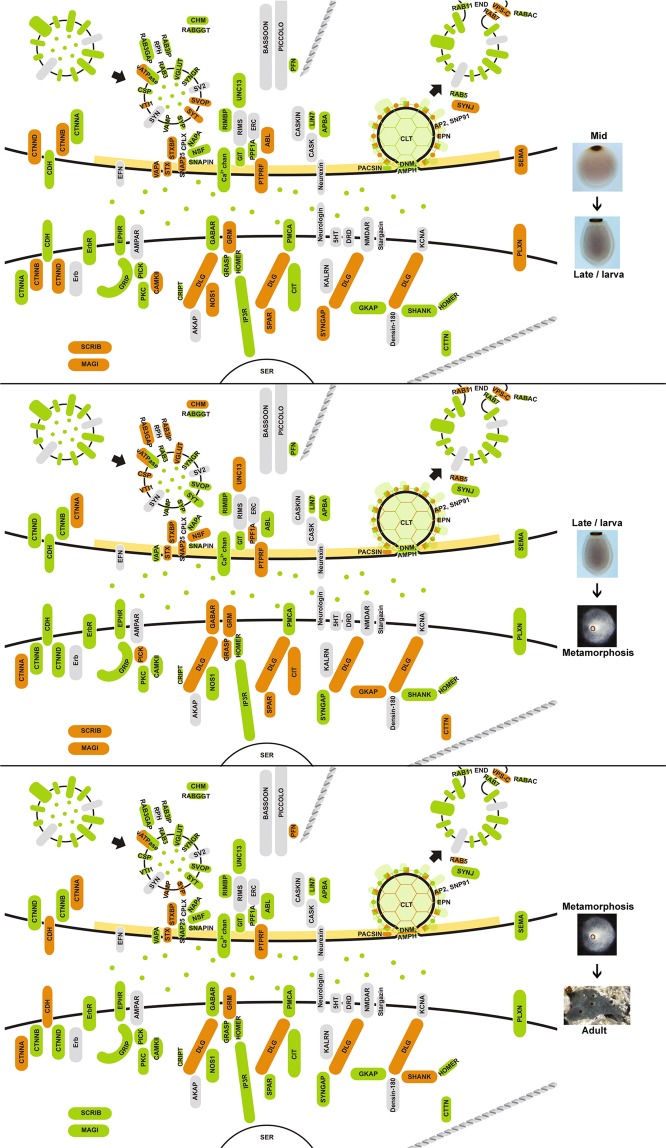
Upregulated synaptic genes during *Amphimedon queenslandica* larval development, metamorphosis and in the adult. Significantly upregulated genes were identified by pairwise comparisons between life stages (p-adj < 0.05) and are coloured orange. Green, orthologues of synaptic genes in *Amphimedon*; grey, genes not present in *Amphimedon*; yellow, active zone.

The majority of proteins involved in the endocytosis pathway in humans^58^, including genes not associated with the synapse, are present in the *Amphimedon* genome (Supplementary Fig. 2). A subset of these genes are tightly co-expressed at high levels in choanocytes (AMPH, AP2B1, AP2M1, AP2S1, DNM1, DNM2, EPN1, RAB5C; lime module, Figs 3 and 5) and metamorphosing and adult life cycle stages (AP2A, AP2B, AP2M, CLTB, CLTC, DNM1, DNM2, EPN1, EPN2, RAB11B, RAB7A; orange module, Figs 4, 6, 7). Monte-Carlo (MC) samplings show that these gene sets significantly co-express both locally (Chi-square H(2) = 41672, p < 0.001) and developmentally (H(2) = 43616, p < 0.001), with degrees of connectivity (C = 0.590 and 0.514 respectively) comparable to those of synaptic submodules (0.340–0.819; Fig. 1B). Other endocytic genes (AP2A2, AP2B1, AP2M1, CLTB, CTTN, DNM1, DNM2, EPN1, EPN2, PACSIN, RAB5, SNAP91) are also co-expressed and upregulated in choanocytes (olive and green modules, Fig. 3 [H(2) = 36732, p < 0.001]; Fig. 5) and in late development (yellow and red modules, Fig. 4 [H(2) = 38511, p < 0.001]; Figs 6, 7). In total, most *Amphimedon* endocytic genes are developmentally co-expressed with the 11 synaptic genes mapped to the endocytic pathway (Supplementary Fig. 2), suggesting that co-expression of synaptic endocytic genes in *Amphimedon* are part of the endocytosis pathway conserved between sponge and bilaterians.

#### Exocytosis

Genes involved in vesicle exocytosis (SLC17A6, SLC17A7, STX1a, STX1b, STX2, STXBP, SNAP25, NSF, NAPA, UNC13, ATP6V0/V1) are also co-regulated during metamorphosis and in adults (orange module, Fig. 4 [H(2) = 45455, p < 0.001]; Figs 6, 7); these genes are co-expressed with some of the endocytosis genes (orange module, Fig. 4). However, these genes are expressed differentially across cell types, and are not enriched in a single adult cell type in this sponge (Figs 3, 5). Genes encoding the SNARE complex (STX1, STXBP/UNC18, SNAP25), which facilitates the fusion of presynaptic vesicles to the plasma membrane during synaptic transmission^59,60^, are co-expressed and upregulated at metamorphosis (orange module, Fig. 4 [H(2) = 42275, p < 0.001]; Fig. 7) and in choanocytes together with calcium channel subunit and cellular calcium flux regulator (CACNA1, PMCA) (lime module, Fig. 3 [H(2) = 41075, p < 0.001]; Fig. 5). Although the SNARE complex contributes to a viable plasma membrane vesicle “dock”^61^, the gene encoding the vesicle-tethering protein VAMP, which is essential for proper SNARE-mediated vesicle fusion, is not co-expressed or significantly upregulated in choanocytes (Figs 3, 5).

#### Zipper mechanism (phagocytosis)

Another pathway that is enriched in metamorphosis is the zipper mechanism of bacterial invasion of epithelial cells, where invasive bacteria interact with receptors on non-phagocytic host cells to activate signaling cascades leading to cytoskeletal rearrangement and bacteria engulfment (orange module, Figs 4, 6, 7; Supplementary Fig. 2)^62^. Although *Amphimedon* has homologues for most of the curated pathway, co-expressed genes (CDH, CLTB, CLTC, CTNNA, CTTN, DNM1, DNM2 [H(2) = 41596, p < 0.001]) primarily contribute to upstream components of the pathway^63^. Genes of this pathway are expressed across all three cell types (Figs 3, 5; Supplementary Fig. 2).

#### Receptors and the post-synaptic density

Scaffolding and signaling molecules in the post-synaptic density (PSD), a key feature of the synapse, play a major role in clustering receptors and are known to have deep unicellular eukaryote origins^42,64–66^. Genes encoding components of the pathways that mediate neurotransmitter receptor functions, stability and trafficking are co-expressed at significantly high levels in the pinacocyte (CAMK2B, GABBR1, GABBR2, GRIP1, PICK1, PRKCB; purple module, Fig. 3 [H(2) = 39293, p < 0.001]; Fig. 5) and co-regulated in late development (AP2A2, AP2B1, AP2M1, CAMK2B, GABBR1, NSF, PRKCB; orange module, Fig. 4 [H(2) = 39482, p < 0.001]; Figs 6, 7). Genes encoding the core scaffolding proteins in PSD (DLG, SHANK, GRASP) are significantly upregulated in choanocytes (green and lime modules, Figs 3, 5).

### Cell type- and stage-specific co-expression of genes with other synaptic functions

In addition to co-expressed synaptic genes corresponding to functional modules or pathways, we find evidence for the upregulation of small sets of synaptic genes in specific cell types and developmental stages. For instance, in addition to genes encoding endocytosis, exocytosis and scaffolding/adhesion proteins being upregulated in choanocytes, the plasma membrane calcium ATPase (PMCA) and the pore-forming α subunit of calcium channel (CACNA1A) are also upregulated in this cell type (Figs 3, 5), suggesting potential electrochemically mediated activities in these cells. A number of signaling genes (CAMK, GABAR, PICK, PRKC, SYNGAP) are upregulated in pinacocytes. In addition, the trans-synaptic pair semaphorin (SEMA) and its receptor plexin (PLXN), known to regulate cell communication and epithelial morphogenesis beyond axon-specific roles^67^, are also upregulated in pinacocytes and during late embryogenesis (Figs 3–7). Several other signaling genes (EGFR, CAMK, GRM, NOS, SYNGAP) are also upregulated in late embryogenesis (Figs 4, 6, 7), just prior to the swimming larval stage that has a capacity to detect light and other environmental signals^68^, including exogenous cues associated with inductive benthic substrata^69^.

## Discussion

A functional synapse evolved through the exaptation of ancient genes with pre-exisiting non-neuronal functions, and the evolution and diversification of new gene families^36,42,44,46,48,70,71^. Together, ancient and more recently evolved synaptic genes were co-opted into a neuronal gene regulatory network that directed the co-expression of all the components necessary for the transmission and reception of synaptic signals; a single cell could have both these functionalities. In this study, we used developmental and cellular gene expression profiles from the sponge *Amphimedon queenslandica* to determine if there is evidence for the co-regulation of genes comprising the modern animal synapse; co-expression is used as a proxy for co-regulation.

To undertake this analysis, we first reassessed the annotation of synaptic genes in the *Amphimedon* genome by extending previous BLAST-based predictions^10,72^ to incorporate other lines of structural and phylogenetic evidence. Although most of our updated gene annotations support previous ones, we do not find sufficient support for the presence of the serotonin and dopamine receptors reported by Srivastava *et al*.^10^. These GPCRs do not clade with eumetazoan representatives^73^ and are therefore regarded as sponge-specific innovations. We also do not find strong support for voltage-gated potassium channels, kalirin and complexin, which were previously reported as present in *Amphimedon*^10,36,42,65^. In contrast, we confirm the absence of ionotrophic glutmate receptors (iGluR)^10^; iGluRs are present in other sponge species^74,75^, suggesting secondary loss of this receptor family in *A. queenslandica*.

Using 82 transcriptomes from individual embryos, larvae, metamorphosing postlarvae and adults condensed into six developmental stages, and 31 manually isolated pools of adult choanocytes, archeocytes and pinacocytes^53,54^, our analyses markedly expand on previous studies that focused on few life cycle stages and on expression of individual genes in specific cell types^46,48,72,76^, and allow for the identification of genes that are differentially co-expressed in specific cell types or at particular developmental stages. We find that co-expressed synaptic genes in *Amphimedon* largely comprise ancient cellular pathways that predate the divergence of metazoans from unicellular holozoans, including exocytotic, endocytotic, induced-phagocytotic and signaling pathways.

In contrast to the global co-expression of synaptic genes in neural animals^36^, we find that these submodules are often separated in *Amphimedon*, displaying cell type- and developmental stage-specific expression profiles. However, amongst the three cell types analysed for this study, the choanocyte co-expresses the most genes. These are associated with three synaptic submodules that predate the divergence of metazoans from choanoflagellates – endocytosis, vesicle docking, and PSD anchoring and scaffolding proteins – along with genes involved with calcium signaling. These functionalities are consistent with the primary role of choanocytes in phagocytic feeding, nutrient sorting and trafficking, and waste elimination, but also may be related to intercellular communication. The ability of PSD proteins to form a complex scaffold predates neural evolution^46^, hence the upregulation of transcripts encoding both PSD proteins and calcium regulators in choanocytes supports the view that the ancestral PSD served to link calcium signaling to cytoskeletal regulation^42^, although this role in choanocytes remains largely unexplored.

The co-upregulation of these multiple synaptic submodules in choanocytes may suggest that this sponge cell type evolved from an ancestral cell that also gave rise to neurons in other animal lineages, but may also be consistent with the choanocyte being a remnant neuron and thus support the proposal that poriferans have secondarily lost a nervous system^21,25,77–79^. Although these data cannot unequivocally support one scenario over the other, it is worth noting that choanocytes share some features with neurons, including an apical microvillar collar and cilium, basal cytoplasmic projections and a raft of dynamic phagosomes and vesicles, and are known to respond rapidly to external stimuli^56,57^. In contrast, nearly all the synaptic components expressed in choanocytes predate metazoans, thus lending support to the “protosynapse” theory^28^, with vesicle-trafficking modules being an aneural neurosecretory apparatus that has been co-opted early in neural evolution^80–82^.

Other synaptic components are co-upregulated in epithelial pinacocytes, namely genes related to the transduction of external signals. The co-expression of receptor-supporting active zone scaffold and receptor-interacting proteins (GRIP, PICK) further supports this epithelial-like cell type being able to sense and respond to exogenous signals. The co-upregulation of trans-synaptic pair SEMA and PLXN in this epithelial cell type suggests that semaphorin–plexin signaling is involved in non-neural cellular processes such as cell movement, migration and proliferation^67^.

Although most synaptic genes are upregulated during metamorphosis and highly expressed in adults, there are smaller subsets that are upregulated during embryogenesis and in larvae. Analysis of synaptic genes in larvae does not support a synapse-like function in known photo- and chemosensory systems underlying larval swimming behavior and responses to exogenous settlement cues^50,51,69,83^. Larval cell types that may contribute to these sensory systems are known to express individual synaptic genes, including globular cells which expressed PSD genes DLG, GKAP, GRIP, HOMER and CRIPT, proneural transcription factor related to atonal/neurogenin-bHLH gene families, and Notch-Delta signaling, and larval pigment ring cells which express a number of neurogenic transcription factors and signaling ligands^46,48,76,84^. Despite the *A. queenslandica* larva having a number of sensory capabilities, there is little evidence for substantial co-regulation of synaptic genes.

The synaptic submodules that were found to be co-expressed in *A. queenslandica* are comprised of genes present also in choanoflagellates^65,82^. This raises the possibility that cell-level sensory behaviours in sponges are akin, and perhaps homologous, to those observed in unicellular eukaryotes^85–87^, although sponges also exhibit tissue- and organismal-level responses to external stimuli^50,83,88^. The lack of strong support for the integration of synaptic submodules under a common regulatory framework in *A. queenslandica* is consistent with sponges not having an integrated synapse or synapse-like function. Analysis of another aneural lineage of animals, the placozoans, provides a means to compare co-expression within and between genes of synaptic submodules to address the origin – and the potential subsequent loss – of neurons at the base of the animal kingdom. Interestingly, however, recent single cell RNA-Seq analysis did not detect strong support for neural gene co-expression in the ctenophore *Mnemiopsis leidyi*^89^, suggesting that module analysis may not adequately resolve the earliest metazoan cladogenic events.

## Conclusions

Analysis of developmental and cell type-specific expression of orthologues of genes encoding human synaptic proteins in *Amphimedon queenslandica* does not find evidence for a near-complete synapse in this sponge. Thus sensory systems and intercellular signaling in this sponge appear to function without synapse-like capabilities. Ancient submodules that comprise the modern synaptome are expressed in specific cell types and life cycle stages, which is consistent with sponges using these submodules as in other eukaryotes. However, the enrichment of multiple submodules and other synaptic genes in choanocytes (i.e. vesicle trafficking, scaffolding, and calcium signaling) suggests the common ancestor of sponges and bilaterians may have possessed a protosynapse involved in localized intercellular communication using exo- and endocytosis.

## Materials and Methods

### Identification of orthologues of synaptic genes in *Amphimedon queenslandica*

A list of synaptic genes was compiled from the canonical human synapse^35,90–95^. These genes partake in the following functions: (i) vesicle exocytosis (including synaptic vesicle surface proteins and vesicle docking machinery); (ii) vesicle recycling via clathrin-mediated endocytosis; (iii) signal transduction (including membrane receptors and some adhesion proteins); (iv) active zone scaffolding; and (v) post-synaptic scaffolding (Fig. 1).

Orthologues of these synaptic genes were identified from the latest version of the *Amphimedon queenslandica* genome, Aqu2.1^10,96^ by: (i) using the human peptide sequences (downloaded from UniProtKB) in a local BLAST at a cut-off value of 1e^−06^; (ii) undertaking a reciprocal-BLAST of potential *A. queenslandica* sequences back to NCBI^97^, with a criterion that at least three of the top five hits must be the relevant synaptic protein; and (iii) examining domain arrangement of candidates with Pfam^98^ and HMMER^99^, with identified domains retained at a cut-off value of 1e^−03^. When putative orthologues had uncertain hits and domain variations between invertebrates and vertebrates, hidden Markov models (HMM) were re-built in-house using only invertebrate sequences, and the putative orthologues reassessed. For some families — dopamine/serotonin receptors, metabotropic glumate receptors (mGluR), γ-aminobutyric acid receptors (GABAR), membrane-associated guanylate kinases (MAGUK), Ras GTPases, cadherins and ion channels — additional information was taken into consideration, including conserved motifs, and structural, functional and phylogenetic analyses^73,78,93,100–120^. The domain arrangements of all *Amphimedon* synaptic were collated and presented using DoMosaics v1.0^121^.

*Amphimedon* synaptic genes identified as above were entered into the interactome databases BioGrid^122^, STRING^123^ and APID^124^ to retrieve evidence-based interactions documented for corresponding orthologues in *Homo sapiens*. All interactions were visualised in Cytoscape v.3.4.0^125^, with duplicate edges, directionality and self-interactions removed. Interactomes from the three databases were non-redundantly merged to produce a “synaptome”, representing all known protein-protein interactions within a functional bilaterian synapse (Fig. 1). The clustering coefficient C, where larger values are indicative of modularity in real-world networks^126–128^, was generated from Cytoscape’s in-built network analysis for each module.

### CEL-Seq RNA datasets

Transcriptomes of *Amphimedon* were previously sequenced and processed using CEL-Seq2^129^ for choanocyte, archeocyte and pinacocyte cell types isolated (under the microscope with a micromanipulator) from adults^53,130^, and CEL-Seq^131^ for 82 developmental samples (whole animals) including embryonic, larval, postlarval, juvenile and adult stages^52,132^ (Table 1). For developmental transcriptomes, the “basic linear index determination of transcriptomes” (BLIND) method was performed^52^ on the 82 developmental samples, which allowed classification into six stages with strong within-group correlation: cleavage; early embryogenesis; mid-embryogenesis; late embryogenesis/larval development; metamorphosis; and adult. These stages were used to compare developmental gene expression profiles (Table 1). Genes with overall CEL-Seq read counts of less than 50 across the three cell types, or 100 across the 82 developmental stages, were discarded. Counts were variance stabilizing transformed (vst) using the Bioconductor package DESeq2^133^ and subjected to principal component analysis (PCA) in R to visualise differences in transcriptome profiles across sample types.

**Table 1:** Developmental and cell-type sampling of *Amphimedon queenslandica* for transcriptome sequencing, by life stages^52^ and cell types^53^.

	Life stage/cell type	No. of samples	Sampling notes
Life stage	Cleavage	7	Individual embryos
	Early embryogenesis	6	Individual embryos
	Mid embryogenesis	19	Individual embryos
	Late embryogenesis/Larva	26	Individual embryos & larvae
	Metamorphosis	18	Individual postlarvae
	Adult	6	Individual sponge biopsies
Cell type	Choanocyte	15 (from 3 animals)	Chambers, 40–60 cells
	Archaeocyte	15 (from 3 animals)	Pools of 5–6 cells
	Pinacocyte	9 (from 3 animals)	Pools of 5–6 cells

### Analysis of functional modules via pathway mapping

Cell type and developmental expression heatmaps were generated with the R packages pheatmap^134^ and RColorBrewer^135^, using the complete linkage method to cluster expression profiles. Genes were classified into 10 (approximate and arbitrary) clades based on expression profile similarities; each profile group was assigned a unique colour. For each group (colour module), all within-group gene interactions were determined using the human-based synaptome built in Cytoscape. Non-interacting genes were removed from the module network. Each module with more than three interacting genes (nodes) was mapped to curated human pathways using the Cytoscape plugin ReactomeFIViz^136^, incorporating data from Reactome^137^, KEGG^58^ and Panther^138^. Filtering was set at a false discovery rate (FDR) of <0.05 and a p-value of < 0.05. Schematic diagrams of selected pathways of interest were downloaded directly from corresponding databases.

### Co-expression validation via Monte Carlo sampling

Co-expressing genes were statistically validated by Monte Carlo (MC) sampling^139^ over 10,000 dendrograms generated by genes of interest (GOIs) and randomly selected Aqu2.1 protein coding genes, with total number of selected genes being equal to that in the original dendrogram generated for synaptic genes. The number of clades GOIs appear in were contrasted against that of five sets of randomly selected control genes (GOCs) sampled in the same manner over 10,000 runs, using the Kruskal-Wallis test and visualising in boxplots (Supplementary Fig. 3). Dunn’s test is used as post-hoc comparison between selected pairs of gene sets to establish signifcant difference. Confidence cut-off is set at p = 0.05.

For selected clusters of co-expressing genes, clustering coefficient C was generated (as described above for the bilaterian synapse). The significance of node connectivities in this cluster is then tested by MC sampling with 10,000 sub-networks induced from an equal number of randomly selected nodes of the synaptome. A cluster is confirmed as a functional submodule of the synapse if the number of interacting edges is within top 5 percentile of the distribution of edge numbers in the 10,000 randomly induced subgraphs (p < 0.05).

All analyses are performed in R. Scripts for analyses are deposited on Github (https://github.com/AquSensory/SciRep2019).

### Differential gene expression analyses

Differential gene expression analyses were performed using the Bioconductor package DESeq2^133^ in R. Differentially expressed genes (DEGs) were extracted by conducting pairwise comparisons between each cell type and between each pair of consecutive developmental stages (p-adj < 0.05). DEGs for each cell type and stage comparison were manually mapped to a custom-made synapse figure to help visualise the synaptic usage of upregulated genes.

## Supplementary information

Supplementary Information Total

## Data Availability

The datasets generated during the current study are available in the Github repository, https://github.com/AquSensory/SciRep2019.
