# Supplementary material for: Co-expression of synaptic genes in the sponge Amphimedon queenslandica uncovers ancient neural submodules
Source: Sci Rep. 2019 Oct 31;9:15781. doi: 10.1038/s41598-019-51282-x (PMC6823388; doi:10.1038/s41598-019-51282-x)

Co-expression of synaptic genes in the sponge *Amphimedon queenslandica* uncovers ancient neural submodules

Eunice Wong, Jan Mölter, Victor Anggono, Sandie M. Degnan and Bernard M. Degnan

Supplementary Files

Supplementary Table 1. Enriched pathways in *Amphimedon* developmental and cell type expression profiles. Expression modules with more than three interacting nodes are mapped to curated pathways in *Homo sapiens* documented by Reactome[137], KEGG[58] and Panther[138] (FDR < 0.05, p < 0.05).

Supplementary Fig. 1. Orthologues of synaptic genes in *Amphimedon queenslandica*. Genome assembly Aqu2.1[96] is used for extraction of orthologues. Protein domain arrangements are shown in comparison to those of corresponding orthologues in *Homo sapiens* (grey-shaded).

Supplementary Fig. 2. Developmental expression profile of *Amphimedon* genes mapped to the KEGG[58] endocytosis and bacterial invasion of epithelial cells pathway. Genes of the (**A-B**) KEGG endocytosis, **(C-E)** bacterial invasion of epithelial cells pathways. Genes of the **A** KEGG endocytosis pathway and the **C** KEGG bacterial invasion of epithelial cells pathway are enriched in co-expression modules of *Amphimedon* synaptic genes (Figs 3, 4). *Amphimedon* possess homologues of the majority of genes coding both pathways in *Homo sapiens*. Developmental expression profiles of the orthologues coding the **B** endocytosis pathway and the **D** bacterial invasion of epithelial cells pathway are largely developmentally

co-regulated (clades consisting the mapped synaptic genes are shaded grey), suggesting that both pathways are conserved in sponge. (**E**) Developmentally co-expressing genes of the bacterial invasion of epithelial cells pathway (grey clade in **D**) are expressed in all three cell types. Green, genes with *Amphimedon* orthologues; blue, genes without *Amphimedon* orthologues; orange, synaptic genes mapped to the corresponding pathway (see Figs 3, 4).

Supplementary Fig. 3. Validation of co-expressing gene sets via Monte Carlo sampling. Boxplots indicate range of number of clades each gene set of interest (GOI) is observed in when sampled over 10,000 developmental / cell type specific expression dendrograms with randomly selected Aqu2.1 protein coding genes. This is contrasted against five sets of randomly selected control genes sampled in the same manner. Boxes show the interquartile range (IQR) and whiskers extend to 1.5 IQR; bold line and circles represent median and outliers respectively. Kruskal-Wallis test and Dunn's post-hoc test confirm all differences between GOI and controls are significant.

Supplementary Table 1.

Module (cell type)	Pathway	Ratio of protein in Gene Set	No. of proteins in geneset	Protein frm network (>3)	P-value (<0.05)	FDR (<0.05)	Nodes
Ol	Synaptic vesicle cycle(K)	0.0058	63	3	1.93E-06	8.50E-05	STX1B,CLTC,ATP6V1E1
	Phagosome(K)	0.0142	154	3	2.79E-05	6.13E-04	STX12,ATP6V1E1,RAB7A
	MHC class II antigen presentation(R)	0.0082	89	2	6.60E-04	9.24E-03	CLTC,RAB7A
	Metabotropic glutamate receptor group III pathway(P)	0.0006	6	1	2.76E-03	0.0303	STX1B
	Metabotropic glutamate receptor group II pathway(P)	0.0008	9	1	4.13E-03	0.0309	STX1B
	Endocytosis(K)	0.0239	260	2	5.46E-03	0.0309	CLTC,RAB7A
	Synaptic_vesicle_trafficking(P)	0.0012	13	1	5.97E-03	0.0309	STX1B
	LGI-ADAM interactions(R)	0.0013	14	1	6.42E-03	0.0309	STX1B
	Collecting duct acid secretion(K)	0.0025	27	1	0.0124	0.0309	ATP6V1E1
	ROS, RNS production in phagocytes(R)	0.0029	32	1	0.0146	0.0309	ATP6V1E1
	FAS (CD95) signaling pathway(N)	0.0029	32	1	0.0146	0.0309	CLTC
	IL8- and CXCR2-mediated signaling events(N)	0.0031	34	1	0.0155	0.0309	RAB7A
	SNARE interactions in vesicular transport(K)	0.0031	34	1	0.0155	0.0309	STX1B
	Arf6 trafficking events(N)	0.0031	34	1	0.0155	0.0309	CLTC
	Iron uptake and transport(R)	0.0035	38	1	0.0174	0.0309	ATP6V1E1
	TBC/RABGAPs(R)	0.0039	42	1	0.0192	0.0309	RAB7A
	trans-Golgi Network Vesicle Budding(R)	0.0041	45	1	0.0205	0.0309	CLTC
	Endocrine and other factor-regulated calcium reabsorption(K)	0.0043	47	1	0.0214	0.0309	CLTC
	Posttranslational regulation of adherens junction stability and dissassembly(N)	0.0044	48	1	0.0219	0.0309	RAB7A
	Vibrio cholerae infection(K)	0.0047	51	1	0.0232	0.0309	ATP6V1E1
	Parkinson disease(P)	0.0056	61	1	0.0278	0.0309	STX12
	IL12-mediated signaling events(N)	0.0056	61	1	0.0278	0.0309	RAB7A
	Epithelial cell signaling in Helicobacter pylori infection(K)	0.0063	68	1	0.0309	0.0309	ATP6V1E1
	RAB GEFs exchange GTP for GDP on RABs(R)	0.0067	73	1	0.0331	0.0331	RAB7A
	Bacterial invasion of epithelial cells(K)	0.0072	78	1	0.0354	0.0354	CLTC
	L1CAM interactions(R)	0.0075	82	1	0.0372	0.0372	CLTC
	Salmonella infection(K)	0.0079	86	1	0.0389	0.0389	RAB7A
	Rheumatoid arthritis(K)	0.0083	90	1	0.0407	0.0407	ATP6V1E1
	Lipid digestion, mobilization, and transport(R)	0.0084	91	1	0.0412	0.0412	CLTC
	Amoebiasis(K)	0.009	98	1	0.0443	0.0443	RAB7A
	Heterotrimeric G-protein signaling pathway-Gq alpha and Go alpha mediated pathway(P)	0.0099	108	1	0.0487	0.0487	CLTC
Gr	Synaptic vesicle cycle(K)	0.0058	63	7	7.88E-15	2.84E-13	ATP6V0B,CLTB,AP2A2,ATP6V0C,ATP6V1D,ATP6V1C1, ATP6V0A1
	ROS, RNS production in phagocytes(R)	0.0029	32	5	2.76E-11	4.97E-10	ATP6V0B,ATP6V0C,ATP6V1D,ATP6V1C1,ATP6V0A1
	Iron uptake and transport(R)	0.0035	38	5	6.51E-11	7.81E-10	ATP6V0B,ATP6V0C,ATP6V1D,ATP6V1C1,ATP6V0A1
	Vibrio cholerae infection(K)	0.0047	51	5	2.82E-10	2.54E-09	ATP6V0B,ATP6V0C,ATP6V1D,ATP6V1C1,ATP6V0A1
	Epithelial cell signaling in Helicobacter pylori infection(K)	0.0063	68	5	1.18E-09	8.29E-09	ATP6V0B,ATP6V0C,ATP6V1D,ATP6V1C1,ATP6V0A1
	Collecting duct acid secretion(K)	0.0025	27	4	4.75E-09	2.39E-08	ATP6V0C,ATP6V1D,ATP6V1C1,ATP6V0A1
	Rheumatoid arthritis(K)	0.0083	90	5	4.78E-09	2.39E-08	ATP6V0B,ATP6V0C,ATP6V1D,ATP6V1C1,ATP6V0A1
	Oxidative phosphorylation(K)	0.0122	133	5	3.32E-08	1.33E-07	ATP6V0B,ATP6V0C,ATP6V1D,ATP6V1C1,ATP6V0A1
	Phagosome(K)	0.0142	154	5	6.87E-08	2.75E-07	ATP6V0B,ATP6V0C,ATP6V1D,ATP6V1C1,ATP6V0A1
	Ion channel transport(R)	0.0156	169	5	1.09E-07	3.26E-07	ATP6V0B,ATP6V0C,ATP6V1D,ATP6V1C1,ATP6V0A1
	Signaling by Insulin receptor(R)	0.0255	277	5	1.24E-06	3.73E-06	ATP6V0B,ATP6V0C,ATP6V1D,ATP6V1C1,ATP6V0A1
	Lysosome(K)	0.0113	123	4	1.98E-06	5.93E-06	ATP6V0B,CLTB,ATP6V0C,ATP6V0A1
	Neutrophil degranulation(R)	0.0362	393	4	1.86E-04	3.72E-04	AP2A2,ATP6V0C,ATP6V1D,ATP6V0A1
	Tuberculosis(K)	0.0165	179	3	3.48E-04	6.97E-04	ATP6V0B,ATP6V0C,ATP6V0A1
	Endocrine and other factor-regulated calcium reabsorption(K)	0.0043	47	2	6.60E-04	1.32E-03	CLTB,AP2A2
	Interactions of neurexins and neuroligins at synapses(R)	0.0046	50	2	7.46E-04	1.49E-03	DLG2,SHANK2
	Metabolic pathways(K)	0.1165	1266	5	1.80E-03	3.60E-03	ATP6V0B,ATP6V0C,ATP6V1D,ATP6V1C1,ATP6V0A1
	Clathrin-mediated endocytosis(R)	0.0108	117	2	3.97E-03	4.24E-03	CLTB,AP2A2
	Huntington disease(P)	0.0111	121	2	4.24E-03	4.24E-03	CLTB,AP2A2
	mTOR signaling pathway(K)	0.0142	154	2	6.77E-03	6.77E-03	ATP6V1D,ATP6V1C1
	Huntington's disease(K)	0.0178	193	2	0.0105	0.0105	CLTB,AP2A2
	Arf1 pathway(N)	0.0018	20	1	0.0164	0.0164	CLTB
	Endocytosis(K)	0.0239	260	2	0.0184	0.0184	CLTB,AP2A2
	adp-ribosylation factor(B)	0.0026	28	1	0.023	0.023	CLTB
Li	Synaptic vesicle cycle(K)	0.0058	63	11	1.11E-16	1.99E-14	SNAP25,CACNA1A,AP2S1,ATP6V0E2,AP2M1,ATP6V1F, AP2B1,DNM1,DNM2,STX1B,ATP6V0D1
	Clathrin-mediated endocytosis(R)	0.0108	117	7	9.22E-09	8.20E-07	AP2S1,PACSIN2,AP2M1,AP2B1,EPN1,DNM2,AMPH
	Endocrine and other factor-regulated calcium reabsorption(K)	0.0043	47	5	9.23E-08	5.02E-06	AP2S1,AP2M1,AP2B1,DNM1,DNM2
	Endocytosis(K)	0.0239	260	8	1.14E-07	5.02E-06	RAB5C,AP2S1,AP2M1,AP2B1,DNM1,EPN1,DNM2,AMPH
	Bacterial invasion of epithelial cells(K)	0.0072	78	5	1.11E-06	3.87E-05	CDH1,CTNNA1,DNM1,DNM2,CTNNB1
	Arf6 trafficking events(N)	0.0031	34	4	1.36E-06	3.93E-05	CDH1,CTNNA1,DNM2,CTNNB1
	Posttranslational regulation of adherens junction stability and dissassembly(N)	0.0044	48	4	5.27E-06	1.32E-04	CDH1,CTNNA1,DNM2,CTNNB1
	E-cadherin signaling in keratinocytes(N)	0.0019	21	3	1.81E-05	3.99E-04	CDH1,CTNNA1,CTNNB1
	Adherens junction(K)	0.0066	72	4	2.56E-05	4.87E-04	PTPRF,CDH1,CTNNA1,CTNNB1
	Collecting duct acid secretion(K)	0.0025	27	3	3.82E-05	6.50E-04	ATP6V0E2,ATP6V1F,ATP6V0D1
	Nectin adhesion pathway(N)	0.0028	30	3	5.22E-05	7.57E-04	CDH1,CTNNA1,CTNNB1
	EPH-Ephrin signaling(R)	0.0082	89	4	5.82E-05	7.57E-04	AP2S1,AP2M1,AP2B1,DNM1
	MHC class II antigen presentation(R)	0.0082	89	4	5.82E-05	7.57E-04	AP2S1,AP2M1,AP2B1,DNM2
	ROS, RNS production in phagocytes(R)	0.0029	32	3	6.31E-05	7.57E-04	ATP6V0E2,ATP6V1F,ATP6V0D1
	SNARE interactions in vesicular transport(K)	0.0031	34	3	7.54E-05	8.30E-04	STX16,VTI1A,STX1B
	Internalization of ErbB1(N)	0.0032	35	3	8.22E-05	8.93E-04	DNM1,EPN1,AMPH
	E-cadherin signaling in the nascent adherens junction(N)	0.0033	36	3	8.93E-05	8.93E-04	CDH1,CTNNA1,CTNNB1
	Iron uptake and transport(R)	0.0035	38	3	1.05E-04	9.42E-04	ATP6V0E2,ATP6V1F,ATP6V0D1
	Stabilization and expansion of the E-cadherin adherens junction(N)	0.0039	42	3	1.40E-04	1.13E-03	CDH1,CTNNA1,CTNNB1
	Beta-catenin independent WNT signaling(R)	0.0103	112	4	1.41E-04	1.13E-03	AP2S1,AP2M1,AP2B1,CTNNB1
	Vibrio cholerae infection(K)	0.0047	51	3	2.48E-04	1.98E-03	ATP6V0E2,ATP6V1F,ATP6V0D1
	Endometrial cancer(K)	0.0048	52	3	2.62E-04	2.05E-03	CDH1,CTNNA1,CTNNB1
	RAC1 signaling pathway(N)	0.005	54	3	2.93E-04	2.05E-03	CDH1,CTNNA1,CTNNB1
	endocytotic role of ndk phosphins and dynamin(B)	0.0012	13	2	4.56E-04	2.75E-03	AP2M1,EPN1
	Synaptic_vesicle_trafficking(P)	0.0012	13	2	4.56E-04	2.75E-03	SNAP25,STX1B
	Signaling events mediated by VEGFR1 and VEGFR2(N)	0.0058	63	3	4.58E-04	2.75E-03	CTNNA1,DNM2,CTNNB1
	Phagosome(K)	0.0142	154	4	4.70E-04	2.82E-03	RAB5C,ATP6V0E2,ATP6V1F,ATP6V0D1
	Epithelial cell signaling in Helicobacter pylori infection(K)	0.0063	68	3	5.72E-04	3.32E-03	ATP6V0E2,ATP6V1F,ATP6V0D1
	CDC42 signaling events(N)	0.0064	70	3	6.22E-04	3.32E-03	CDH1,CTNNA1,CTNNB1
	Ion channel transport(R)	0.0156	169	4	6.65E-04	3.32E-03	ATP6V0E2,ATP6V1F,ATP2B2,ATP6V0D1
	Signaling events mediated by Hepatocyte Growth Factor Receptor (c-Met)(N)	0.0074	80	3	9.14E-04	4.57E-03	CDH1,CTNNA1,CTNNB1
	L1CAM interactions(R)	0.0075	82	3	9.81E-04	4.90E-03	AP2S1,AP2M1,AP2B1
	Rheumatoid arthritis(K)	0.0083	90	3	1.28E-03	6.14E-03	ATP6V0E2,ATP6V1F,ATP6V0D1
	Lipid digestion, mobilization, and transport(R)	0.0084	91	3	1.32E-03	6.14E-03	AP2S1,AP2M1,AP2B1
	Myogenesis(R)	0.0022	24	2	1.53E-03	6.14E-03	CTNNA1,CTNNB1
	Rap1 signaling pathway(K)	0.0195	212	4	1.54E-03	6.14E-03	CDH1,CTNNB1,PFN4,PFN2

	Cadherin signaling pathway(P)	0.0092	100	3	1.73E-03	6.92E-03	CDH1,CTNNA1,CTNNB1
	Thyroid cancer(K)	0.0027	29	2	2.22E-03	8.87E-03	CDH1,CTNNB1
	Alzheimer disease-presenilin pathway(P)	0.0102	111	3	2.32E-03	9.30E-03	CDH1,CTNNA1,CTNNB1
	N-cadherin signaling events(N)	0.003	33	2	2.86E-03	0.0114	CTNNA1,CTNNB1
	Oxidative phosphorylation(K)	0.0122	133	3	3.86E-03	0.0154	ATP6V0E2,ATP6V1F,ATP6V0D1
	Neurotransmitter Receptor Binding And Downstream Transmission In The Postsynaptic Cell(R)	0.0123	134	3	3.94E-03	0.0158	AP2S1,AP2M1,AP2B1
	PAR1-mediated thrombin signaling events(N)	0.004	43	2	4.78E-03	0.0174	DNM1,DNM2
	Apoptotic execution phase(R)	0.0043	47	2	5.67E-03	0.0174	CDH1,CTNNB1
	Hippo signaling pathway(K)	0.0142	154	3	5.80E-03	0.0174	CDH1,CTNNA1,CTNNB1
	AlphaE beta7 integrin cell surface interactions(N)	0.0003	3	1	7.15E-03	0.0215	CDH1
	Pathogenic Escherichia coli infection(K)	0.0051	55	2	7.68E-03	0.023	CDH1,CTNNB1
	Metabotropic glutamate receptor group I pathway(P)	0.0004	4	1	9.53E-03	0.0286	HOMER1
	Shigellosis(K)	0.006	65	2	0.0106	0.0317	PFN4,PFN2
	Huntington's disease(K)	0.0178	193	3	0.0107	0.0322	AP2S1,AP2M1,AP2B1
	Cell junction organization(R)	0.0064	70	2	0.0122	0.0365	CDH1,CTNNA1
	Arrhythmogenic right ventricular cardiomyopathy (ARVC)(K)	0.0066	72	2	0.0128	0.0385	CTNNA1,CTNNB1
	Metabotropic glutamate receptor group III pathway(P)	0.0006	6	1	0.0143	0.0426	STX1B
	Regulation of nuclear beta catenin signaling and target gene transcription(N)	0.0074	80	2	0.0157	0.0426	CDH1,CTNNB1
	Depolarization of the Presynaptic Terminal Triggers the Opening of Calcium Channels(R)	0.0006	7	1	0.0166	0.0426	CACNA1A
	sumoylation as a mechanism to modulate ctbp-dependent gene responses(B)	0.0006	7	1	0.0166	0.0426	CDH1
	Salmonella infection(K)	0.0079	86	2	0.0179	0.0426	PFN4,PFN2
	Integration of energy metabolism(R)	0.0085	92	2	0.0204	0.0426	SNAP25,CACNA1A
	Fc gamma R-mediated phagocytosis(K)	0.0086	93	2	0.0208	0.0426	DNM2,AMPH
	Effects of Botulinum toxin(N)	0.0008	9	1	0.0213	0.0426	SNAP25
	Metabotropic glutamate receptor group II pathway(P)	0.0008	9	1	0.0213	0.0426	STX1B
	Gap junction trafficking and regulation(R)	0.0008	9	1	0.0213	0.0426	AP2M1
	5HT3 type receptor mediated signaling pathway(P)	0.0009	10	1	0.0237	0.0473	SNAP25
Te	Synaptic vesicle cycle(K)	0.0058	63	2	2.00E-04	6.37E-03	STX1B,CLTC
	MHC class II antigen presentation(R)	0.0082	89	2	3.98E-04	6.37E-03	CLTC,RAB7A
	Phagosome(K)	0.0142	154	2	1.18E-03	0.013	STX12,RAB7A
	Metabotropic glutamate receptor group III pathway(P)	0.0006	6	1	2.21E-03	0.0166	STX1B
	Metabotropic glutamate receptor group II pathway(P)	0.0008	9	1	3.31E-03	0.0166	STX1B
	Endocytosis(K)	0.0239	260	2	3.33E-03	0.0166	CLTC,RAB7A
	Synaptic_vesicle_trafficking(P)	0.0012	13	1	4.78E-03	0.0191	STX1B
	LGI-ADAM interactions(R)	0.0013	14	1	5.14E-03	0.0206	STX1B
	FAS (CD95) signaling pathway(N)	0.0029	32	1	0.0117	0.0223	CLTC
	IL8- and CXCR2-mediated signaling events(N)	0.0031	34	1	0.0125	0.0223	RAB7A
	SNARE interactions in vesicular transport(K)	0.0031	34	1	0.0125	0.0223	STX1B
	Arf6 trafficking events(N)	0.0031	34	1	0.0125	0.0223	CLTC
	TBC/RABGAPs(R)	0.0039	42	1	0.0154	0.0223	RAB7A
	trans-Golgi Network Vesicle Budding(R)	0.0041	45	1	0.0165	0.0223	CLTC
	Endocrine and other factor-regulated calcium reabsorption(K)	0.0043	47	1	0.0172	0.0223	CLTC
	Posttranslational regulation of adherens junction stability and dissassembly(N)	0.0044	48	1	0.0176	0.0223	RAB7A
	Parkinson disease(P)	0.0056	61	1	0.0223	0.0223	STX12
	IL12-mediated signaling events(N)	0.0056	61	1	0.0223	0.0223	RAB7A
	RAB GEFs exchange GTP for GDP on RABs(R)	0.0067	73	1	0.0266	0.0266	RAB7A
	Bacterial invasion of epithelial cells(K)	0.0072	78	1	0.0284	0.0284	CLTC
	L1CAM interactions(R)	0.0075	82	1	0.0298	0.0298	CLTC
	Salmonella infection(K)	0.0079	86	1	0.0313	0.0313	RAB7A
	Lipid digestion, mobilization, and transport(R)	0.0084	91	1	0.0331	0.0331	CLTC
	Amoebiasis(K)	0.009	98	1	0.0356	0.0356	RAB7A
	Heterotrimeric G-protein signaling pathway-Gq alpha and Go alpha mediated pathway(P)	0.0099	108	1	0.0392	0.0392	CLTC
	Beta-catenin independent WNT signaling(R)	0.0103	112	1	0.0406	0.0406	CLTC
	Clathrin-mediated endocytosis(R)	0.0108	117	1	0.0424	0.0424	CLTC
	Lysosome(K)	0.0113	123	1	0.0445	0.0445	CLTC
Na	Collecting duct acid secretion(K)	0.0025	27	2	1.85E-05	3.29E-04	ATP6V1A,ATP6V1B1
	ROS, RNS production in phagocytes(R)	0.0029	32	2	2.60E-05	3.29E-04	ATP6V1A,ATP6V1B1
	Iron uptake and transport(R)	0.0035	38	2	3.66E-05	3.29E-04	ATP6V1A,ATP6V1B1
	Vibrio cholerae infection(K)	0.0047	51	2	6.59E-05	3.95E-04	ATP6V1A,ATP6V1B1
	Synaptic vesicle cycle(K)	0.0058	63	2	1.00E-04	4.68E-04	ATP6V1A,ATP6V1B1
	Epithelial cell signaling in Helicobacter pylori infection(K)	0.0063	68	2	1.17E-04	4.68E-04	ATP6V1A,ATP6V1B1
	Rheumatoid arthritis(K)	0.0083	90	2	2.05E-04	6.14E-04	ATP6V1A,ATP6V1B1
	Oxidative phosphorylation(K)	0.0122	133	2	4.46E-04	1.19E-03	ATP6V1A,ATP6V1B1
	Phagosome(K)	0.0142	154	2	5.97E-04	1.19E-03	ATP6V1A,ATP6V1B1
	mTOR signaling pathway(K)	0.0142	154	2	5.97E-04	1.19E-03	ATP6V1A,ATP6V1B1
	Ion channel transport(R)	0.0156	169	2	7.18E-04	1.44E-03	ATP6V1A,ATP6V1B1
	Signaling by Insulin receptor(R)	0.0255	277	2	1.92E-03	3.83E-03	ATP6V1A,ATP6V1B1
	5HT3 type receptor mediated signaling pathway(P)	0.0009	10	1	2.76E-03	5.52E-03	VAMP8
	Beta3 adrenergic receptor signaling pathway(P)	0.0019	21	1	5.79E-03	5.79E-03	VAMP8
	Beta2 adrenergic receptor signaling pathway(P)	0.0019	21	1	5.79E-03	5.79E-03	VAMP8
	Oxytocin receptor mediated signaling pathway(P)	0.0019	21	1	5.79E-03	5.79E-03	VAMP8
	5HT4 type receptor mediated signaling pathway(P)	0.0021	23	1	6.34E-03	6.34E-03	VAMP8
	Cortocotropin releasing factor receptor signaling pathway(P)	0.0023	25	1	6.89E-03	6.89E-03	VAMP8
	5HT2 type receptor mediated signaling pathway(P)	0.0024	26	1	7.16E-03	7.16E-03	VAMP8
	Beta1 adrenergic receptor signaling pathway(P)	0.0025	27	1	7.44E-03	7.44E-03	VAMP8
	SNARE interactions in vesicular transport(K)	0.0031	34	1	9.36E-03	9.36E-03	VAMP8
	trans-Golgi Network Vesicle Budding(R)	0.0041	45	1	0.0124	0.0124	VAMP8
	Clathrin-mediated endocytosis(R)	0.0108	117	1	0.032	0.032	VAMP8
	Platelet activation(K)	0.0112	122	1	0.0333	0.0333	VAMP8
	Metabolic pathways(K)	0.1165	1266	2	0.0376	0.0376	ATP6V1A,ATP6V1B1
Sk	Metabotropic glutamate receptor group III pathway(P)	0.0006	6	2	1.83E-06	8.77E-05	STX1B,STX1A
	Metabotropic glutamate receptor group II pathway(P)	0.0008	9	2	4.11E-06	9.87E-05	STX1B,STX1A
	Synaptic_vesicle_trafficking(P)	0.0012	13	2	8.57E-06	1.19E-04	STX1B,STX1A
	LGI-ADAM interactions(R)	0.0013	14	2	9.94E-06	1.19E-04	STX1B,STX1A
	SNARE interactions in vesicular transport(K)	0.0031	34	2	5.85E-05	5.26E-04	STX1B,STX1A
	Synaptic vesicle cycle(K)	0.0058	63	2	2.00E-04	1.60E-03	STX1B,STX1A
	Effects of Botulinum toxin(N)	0.0008	9	1	3.31E-03	0.0199	STX1A
	RAB geranylgeranylation(R)	0.0014	15	1	5.51E-03	0.0219	RAB11B
	tumor suppressor arf inhibits ribosomal biogenesis(B)	0.0017	19	1	6.98E-03	0.0219	ABL1
	Insulin processing(R)	0.0018	20	1	7.34E-03	0.0219	STX1A
	Signaling by Robo receptor(R)	0.0021	23	1	8.44E-03	0.0219	ABL1
	Myogenesis(R)	0.0022	24	1	8.80E-03	0.0219	ABL1
	Lissencephaly gene (LIS1) in neuronal migration and development(N)	0.0026	28	1	0.0103	0.0219	ABL1
	ATM pathway(N)	0.0031	34	1	0.0125	0.0219	ABL1
	Neurotransmitter Release Cycle(R)	0.0037	40	1	0.0146	0.0219	STX1A
	TBC/RABGAPs(R)	0.0039	42	1	0.0154	0.0219	RAB11B

	Vasopressin-regulated water reabsorption(K)	0.004	44	1	0.0161	0.0219	RAB11B
	DNA Double Strand Break Response(R)	0.0041	45	1	0.0165	0.0219	ABL1
	Posttranslational regulation of adherens junction stability and dissassembly(N)	0.0044	48	1	0.0176	0.0219	ABL1
	Validated transcriptional targets of TAp63 isoforms(N)	0.0045	49	1	0.0179	0.0219	ABL1
	Interactions of neurexins and neuroligins at synapses(R)	0.0046	50	1	0.0183	0.0219	STX1A
	Pathogenic Escherichia coli infection(K)	0.0051	55	1	0.0201	0.0219	ABL1
	p53 pathway(N)	0.0052	57	1	0.0208	0.0219	ABL1
	Viral myocarditis(K)	0.0054	59	1	0.0215	0.0219	ABL1
	Neurotrophic factor-mediated Trk receptor signaling(N)	0.0055	60	1	0.0219	0.0219	ABL1
	Regulation of retinoblastoma protein(N)	0.0059	64	1	0.0234	0.0234	ABL1
	Shigellosis(K)	0.006	65	1	0.0237	0.0237	ABL1
	Regulation of Telomerase(N)	0.0063	68	1	0.0248	0.0248	ABL1
	Amphetamine addiction(K)	0.0063	68	1	0.0248	0.0248	STX1A
	Chronic myeloid leukemia(K)	0.0067	73	1	0.0266	0.0266	ABL1
	p73 transcription factor network(N)	0.0069	75	1	0.0273	0.0273	ABL1
	Insulin secretion(K)	0.0078	85	1	0.0309	0.0309	STX1A
	HDR through Homologous Recombination (HR) or Single Strand Annealing (SSA)(R)	0.0078	85	1	0.0309	0.0309	ABL1
	ErbB signaling pathway(K)	0.0081	88	1	0.032	0.032	ABL1
	Integration of energy metabolism(R)	0.0085	92	1	0.0334	0.0334	STX1A
	Factors involved in megakaryocyte development and platelet production(R)	0.0101	110	1	0.0399	0.0399	ABL1
	PDGFR-beta signaling pathway(N)	0.011	120	1	0.0434	0.0434	ABL1
	Neurotrophin signaling pathway(K)	0.0111	121	1	0.0438	0.0438	ABL1
	Cell cycle(K)	0.0114	124	1	0.0449	0.0449	ABL1
	AMPK signaling pathway(K)	0.0114	124	1	0.0449	0.0449	RAB11B
Pu	Neurotransmitter Receptor Binding And Downstream Transmission In The Postsynaptic Cell(R)	0.0123	134	6	1.60E-08	2.83E-06	CAMK2B,GABBR2,GABBR1,PRKCB,GRIP1,PICK1
	Neurotransmitter Release Cycle(R)	0.0037	40	3	2.20E-05	1.93E-03	UNC13B,SYT1,PPFIA2
	Effects of Botulinum toxin(N)	0.0008	9	2	7.15E-05	3.66E-03	UNC13B,SYT1
	Axon guidance(K)	0.0163	177	4	8.32E-05	3.66E-03	CAMK2B,SEMA5B,EPHB1,PLXNA3
	Synaptic_vesicle_trafficking(P)	0.0012	13	2	1.49E-04	5.21E-03	UNC13B,SYT1
	GABAergic synapse(K)	0.0081	88	3	2.25E-04	5.50E-03	GABBR2,GABBR1,PRKCB
	Morphine addiction(K)	0.0084	91	3	2.48E-04	5.50E-03	GABBR2,GABBR1,PRKCB
	nitric oxide signaling pathway(B)	0.0016	17	2	2.54E-04	5.50E-03	PRKCB,NOS1
	Circadian entrainment(K)	0.0088	96	3	2.90E-04	5.50E-03	CAMK2B,PRKCB,NOS1
	vegf hypoxia and angiogenesis(B)	0.0026	28	2	6.82E-04	0.0116	PRKCB,NOS1
	Calcium signaling pathway(K)	0.0167	182	3	1.84E-03	0.0294	CAMK2B,PRKCB,NOS1
	cAMP signaling pathway(K)	0.0184	200	3	2.40E-03	0.0336	CAMK2B,GABBR2,GABBR1
	Long-term depression(K)	0.0055	60	2	3.05E-03	0.0389	PRKCB,NOS1
	Synaptic vesicle cycle(K)	0.0058	63	2	3.36E-03	0.0389	UNC13B,SYT1
	Glioma(K)	0.0061	66	2	3.67E-03	0.0389	CAMK2B,PRKCB
	Long-term potentiation(K)	0.0062	67	2	3.78E-03	0.0389	CAMK2B,PRKCB
	Amphetamine addiction(K)	0.0063	68	2	3.89E-03	0.0389	CAMK2B,PRKCB
	Gastric acid secretion(K)	0.0069	75	2	4.71E-03	0.042	CAMK2B,PRKCB
	Aldosterone synthesis and secretion(K)	0.0075	82	2	5.60E-03	0.042	CAMK2B,PRKCB
	Taste transduction(K)	0.0076	83	2	5.73E-03	0.042	GABBR2,GABBR1
	Insulin secretion(K)	0.0078	85	2	6.00E-03	0.042	CAMK2B,PRKCB
	Neuroactive ligand-receptor interaction(K)	0.0256	278	3	6.05E-03	0.042	GABBR2,GABBR1,GRM3
	Potassium Channels(R)	0.008	87	2	6.28E-03	0.042	GABBR2,GABBR1
	ErbB signaling pathway(K)	0.0081	88	2	6.42E-03	0.042	CAMK2B,PRKCB
	Salivary secretion(K)	0.0083	90	2	6.70E-03	0.042	PRKCB,NOS1
	GnRH signaling pathway(K)	0.0085	92	2	6.99E-03	0.042	CAMK2B,PRKCB
	Inflammatory mediator regulation of TRP channels(K)	0.0091	99	2	8.05E-03	0.0435	CAMK2B,PRKCB
	Estrogen signaling pathway(K)	0.0092	100	2	8.21E-03	0.0435	GABBR2,GABBR1
	Melanogenesis(K)	0.0093	101	2	8.37E-03	0.0435	CAMK2B,PRKCB
	HIF-1 signaling pathway(K)	0.0095	103	2	8.69E-03	0.0435	CAMK2B,PRKCB
	Cardiac conduction(R)	0.0098	107	2	9.35E-03	0.0467	CAMK2B,NOS1
	Heterotrimeric G-protein signaling pathway-Gq alpha and Go alpha mediated pathway(P)	0.0099	108	2	9.52E-03	0.0476	PRKCB,GRM3
	cbl mediated ligand-induced downregulation of egf receptors pathway(B)	0.0006	7	1	9.62E-03	0.0481	PRKCB
La	RAB GEFs exchange GTP for GDP on RABs(R)	0.0067	73	3	3.00E-06	2.10E-05	RAB3GAP2,CHM,RAB3GAP1
	RAB geranylgeranylation(R)	0.0014	15	2	1.90E-05	5.70E-05	CHM,RABGGTB
	Intra-Golgi and retrograde Golgi-to-ER traffic(R)	0.013	141	2	1.64E-03	3.28E-03	RAB3GAP2,RAB3GAP1
	Effects of Botulinum toxin(N)	0.0008	9	1	4.13E-03	4.13E-03	RAB3GAP2
	Signaling events mediated by PRL(N)	0.0021	23	1	0.0105	0.0105	RABGGTB
	Generic Transcription Pathway(R)	0.0455	494	2	0.0188	0.0188	CHM,RABGGTB
	Sphingolipid metabolism(R)	0.0065	71	1	0.0322	0.0322	VAPB
Gy	SNARE interactions in vesicular transport(K)	0.0031	34	2	9.73E-05	1.75E-03	STX5,STX2
	Hippo signaling pathway(K)	0.0142	154	2	1.95E-03	0.0176	DLG1,SCRIB
	Viral carcinogenesis(K)	0.0187	203	2	3.36E-03	0.0202	DLG1,SCRIB
	SALM protein interactions at the synapses(R)	0.0018	20	1	9.17E-03	0.0318	DLG1
	E-cadherin signaling in the nascent adherens junction(N)	0.0033	36	1	0.0165	0.0318	DLG1
	Neurotransmitter Release Cycle(R)	0.0037	40	1	0.0183	0.0318	APBA1
	Interactions of neurexins and neuroligins at synapses(R)	0.0046	50	1	0.0228	0.0318	APBA1
	Alzheimer disease-amyloid secretase pathway(P)	0.0054	59	1	0.0269	0.0318	APBA1
	Synaptic vesicle cycle(K)	0.0058	63	1	0.0287	0.0318	STX2
	CDC42 signaling events(N)	0.0064	70	1	0.0318	0.0318	DLG1
	L1CAM interactions(R)	0.0075	82	1	0.0372	0.0372	DLG1
	T cell receptor signaling pathway(K)	0.0097	105	1	0.0474	0.0474	DLG1

Module (dev'tal)	Pathway	Ratio of protein in Gene Set	No. of proteins in geneset	Protein frm network (>3)	P-value (<0.05)	FDR (<0.05)	Nodes
Re	Synaptic vesicle cycle(K)	0.0058	63	8	1.55E-15	7.93E-14	ATP6V1A,ATP6V0B,STXBP1,STX1B,AP2S1,ATP6V1E1,ATP6V0C,ATP6V1D
	ROS, RNS production in phagocytes(R)	0.0029	32	5	2.79E-10	6.99E-09	ATP6V1A,ATP6V0B,ATP6V1E1,ATP6V0C,ATP6V1D
	Iron uptake and transport(R)	0.0035	38	5	6.57E-10	1.12E-08	ATP6V1A,ATP6V0B,ATP6V1E1,ATP6V0C,ATP6V1D
	Vibrio cholerae infection(K)	0.0047	51	5	2.84E-09	3.41E-08	ATP6V1A,ATP6V0B,ATP6V1E1,ATP6V0C,ATP6V1D
	Epithelial cell signaling in Helicobacter pylori infection(K)	0.0063	68	5	1.18E-08	1.18E-07	ATP6V1A,ATP6V0B,ATP6V1E1,ATP6V0C,ATP6V1D
	Rheumatoid arthritis(K)	0.0083	90	5	4.74E-08	3.32E-07	ATP6V1A,ATP6V0B,ATP6V1E1,ATP6V0C,ATP6V1D
	Oxidative phosphorylation(K)	0.0122	133	5	3.26E-07	1.95E-06	ATP6V1A,ATP6V0B,ATP6V1E1,ATP6V0C,ATP6V1D
	Phagosome(K)	0.0142	154	5	6.69E-07	3.34E-06	ATP6V1A,ATP6V0B,ATP6V1E1,ATP6V0C,ATP6V1D
	Ion channel transport(R)	0.0156	169	5	1.05E-06	5.27E-06	ATP6V1A,ATP6V0B,ATP6V1E1,ATP6V0C,ATP6V1D
	Signaling by Insulin receptor(R)	0.0255	277	5	1.17E-05	4.67E-05	ATP6V1A,ATP6V0B,ATP6V1E1,ATP6V0C,ATP6V1D
	Metabolic pathways(K)	0.1165	1266	5	0.0123	0.0188	ATP6V1A,ATP6V0B,ATP6V1E1,ATP6V0C,ATP6V1D
	Collecting duct acid secretion(K)	0.0025	27	4	2.68E-08	2.14E-07	ATP6V1A,ATP6V1E1,ATP6V0C,ATP6V1D
	SNARE interactions in vesicular transport(K)	0.0031	34	3	8.56E-06	3.42E-05	STX16,VAMP8,STX1B
	Intra-Golgi and retrograde Golgi-to-ER traffic(R)	0.013	141	3	5.67E-04	1.70E-03	STX16,RAB3GAP2,RAB3GAP1
	mTOR signaling pathway(K)	0.0142	154	3	7.32E-04	2.20E-03	ATP6V1A,ATP6V1E1,ATP6V1D

	Neutrophil degranulation(R)	0.0362	393	3	0.0103	0.0188	VAMP8,ATP6V0C,ATP6V1D
	Effects of Botulinum toxin(N)	0.0008	9	2	5.32E-05	1.60E-04	STXBP1,RAB3GAP2
	Synaptic_vesicle_trafficking(P)	0.0012	13	2	1.11E-04	3.32E-04	STXBP1,STX1B
	RAB GEFs exchange GTP for GDP on RABs(R)	0.0067	73	2	3.35E-03	0.0101	RAB3GAP2,RAB3GAP1
	Clathrin-mediated endocytosis(R)	0.0108	117	2	8.36E-03	0.0167	VAMP8,AP2S1
	Lysosome(K)	0.0113	123	2	9.20E-03	0.0184	ATP6V0B,ATP6V0C
	Tuberculosis(K)	0.0165	179	2	0.0188	0.0188	ATP6V0B,ATP6V0C
	Metabotropic glutamate receptor group III pathway(P)	0.0006	6	1	7.15E-03	0.0143	STX1B
	Metabotropic glutamate receptor group II pathway(P)	0.0008	9	1	0.0107	0.0188	STX1B
	5HT3 type receptor mediated signaling pathway(P)	0.0009	10	1	0.0119	0.0188	VAMP8
	LGI-ADAM interactions(R)	0.0013	14	1	0.0166	0.0188	STX1B
	Beta2 adrenergic receptor signaling pathway(P)	0.0019	21	1	0.0248	0.0248	VAMP8
	Beta3 adrenergic receptor signaling pathway(P)	0.0019	21	1	0.0248	0.0248	VAMP8
	Oxytocin receptor mediated signaling pathway(P)	0.0019	21	1	0.0248	0.0248	VAMP8
	5HT4 type receptor mediated signaling pathway(P)	0.0021	23	1	0.0272	0.0272	VAMP8
	Cortocotropin releasing factor receptor signaling pathway(P)	0.0023	25	1	0.0295	0.0295	VAMP8
	5HT2 type receptor mediated signaling pathway(P)	0.0024	26	1	0.0307	0.0307	VAMP8
	Beta1 adrenergic receptor signaling pathway(P)	0.0025	27	1	0.0318	0.0318	VAMP8
	Neurotransmitter Release Cycle(R)	0.0037	40	1	0.0468	0.0468	STXBP1
Go	Neurotransmitter Release Cycle(R)	0.0037	40	3	1.39E-05	1.71E-03	SYT1,DNAJC5,APBA1
Or	Synaptic vesicle cycle(K)	0.0058	63	20	1.11E-16	3.04E-14	NAPA,SNAP25,STXBP1,CLTC,CLTB,AP2A2,ATP6V1H, SLC17A6,SLC17A7,STX2,AP2M1,ATP6V1C1,NSF,UNC13B, AP2B1,DNM1,DNM2,STX1B,STX1A,ATP6V1B1
	Endocytosis(K)	0.0239	260	11	1.33E-09	7.36E-08	CLTC,CLTB,AP2A2,AP2M1,AP2B1,EPN1,DNM1,RAB11B, DNM2,EPN2,RAB7A
	Clathrin-mediated endocytosis(R)	0.0108	117	10	1.00E-11	9.11E-10	CLTC,CLTB,AP2A2,AP2M1,AP2B1,EPN1,DNM2,EPN2, CTTN,SYNJ1
	Endocrine and other factor-regulated calcium reabsorption(K)	0.0043	47	8	6.69E-12	9.11E-10	CLTC,CLTB,AP2A2,AP2M1,PRKCB,AP2B1,DNM1,DNM2
	Bacterial invasion of epithelial cells(K)	0.0072	78	7	1.23E-08	5.55E-07	CLTC,CLTB,CDH1,CTNNA1,DNM1,DNM2,CTTN
	Neurotransmitter Receptor Binding And Downstream Transmission In The Postsynaptic Cell(R)	0.0123	134	7	4.72E-07	1.41E-05	CAMK2B,AP2A2,AP2M1,NSF,GABBR1,PRKCB,AP2B1
	MHC class II antigen presentation(R)	0.0082	89	6	7.81E-07	2.11E-05	CLTC,AP2A2,AP2M1,AP2B1,DNM2,RAB7A
	Signalling by NGF(R)	0.0387	421	6	3.67E-03	0.0208	CAMK2B,CLTC,AP2A2,SYNGAP1,AP2M1,AP2B1
	Synaptic_vesicle_trafficking(P)	0.0012	13	5	1.36E-09	7.36E-08	SNAP25,STXBP1,UNC13B,STX1B,STX1A
	Neurotransmitter Release Cycle(R)	0.0037	40	5	3.51E-07	1.19E-05	SNAP25,STXBP1,SLC17A7,UNC13B,STX1A
	Phagosome(K)	0.0142	154	5	2.20E-04	3.34E-03	STX12,ATP6V1H,ATP6V1C1,RAB7A,ATP6V1B1
	Ion channel transport(R)	0.0156	169	5	3.37E-04	3.37E-03	CAMK2B,ATP6V1H,ATP6V1C1,ATP2B2,ATP6V1B1
	Huntington's disease(K)	0.0178	193	5	6.15E-04	4.92E-03	CLTC,CLTB,AP2A2,AP2M1,AP2B1
	Signaling by Insulin receptor(R)	0.0255	277	5	3.01E-03	0.0181	CAMK2B,SYNGAP1,ATP6V1H,ATP6V1C1,ATP6V1B1
	Effects of Botulinum toxin(N)	0.0008	9	4	3.78E-08	1.47E-06	SNAP25,STXBP1,UNC13B,STX1A
	Arf6 trafficking events(N)	0.0031	34	4	7.22E-06	1.59E-04	CLTC,CDH1,CTNNA1,DNM2
	SNARE interactions in vesicular transport(K)	0.0031	34	4	7.22E-06	1.59E-04	STX16,STX2,STX1B,STX1A
	Posttranslational regulation of adherens junction stability and dissassembly(N)	0.0044	48	4	2.77E-05	5.26E-04	CDH1,CTNNA1,DNM2,RAB7A
	L1CAM interactions(R)	0.0075	82	4	2.16E-04	3.34E-03	CLTC,AP2A2,AP2M1,AP2B1
	Insulin secretion(K)	0.0078	85	4	2.47E-04	3.34E-03	CAMK2B,SNAP25,PRKCB,STX1A
	EPH-Ephrin signaling(R)	0.0082	89	4	2.94E-04	3.34E-03	AP2A2,AP2M1,AP2B1,DNM1
	Lipid digestion, mobilization, and transport(R)	0.0084	91	4	3.20E-04	3.37E-03	CLTC,AP2A2,AP2M1,AP2B1
	Heterotrimeric G-protein signaling pathway-Gq alpha and Go alpha mediated pathway(P)	0.0099	108	4	6.08E-04	4.92E-03	CLTC,CLTB,GRM3,PRKCB
	Beta-catenin independent WNT signaling(R)	0.0103	112	4	6.96E-04	5.57E-03	CLTC,AP2A2,AP2M1,AP2B1
	Glutamatergic synapse(K)	0.0105	114	4	7.43E-04	5.94E-03	GRM3,SLC17A6,SLC17A7,PRKCB
	mTOR signaling pathway(K)	0.0142	154	4	2.23E-03	0.0134	ATP6V1H,ATP6V1C1,PRKCB,ATP6V1B1
	endocytotic role of ndk phosphins and dynamin(B)	0.0012	13	3	1.51E-05	3.18E-04	AP2M1,EPN1,SYNJ1
	ROS, RNS production in phagocytes(R)	0.0029	32	3	2.16E-04	3.34E-03	ATP6V1H,ATP6V1C1,ATP6V1B1
	IL8- and CXCR2-mediated signaling events(N)	0.0031	34	3	2.57E-04	3.34E-03	PRKCB,DNM1,RAB7A
	Internalization of ErbB1(N)	0.0032	35	3	2.80E-04	3.34E-03	EPN1,DNM1,SYNJ1
	E-cadherin signaling in the nascent adherens junction(N)	0.0033	36	3	3.04E-04	3.34E-03	CDH1,CTNNA1,CTTN
	Iron uptake and transport(R)	0.0035	38	3	3.56E-04	3.56E-03	ATP6V1H,ATP6V1C1,ATP6V1B1
	PAR1-mediated thrombin signaling events(N)	0.004	43	3	5.09E-04	4.58E-03	PRKCB,DNM1,DNM2
	trans-Golgi Network Vesicle Budding(R)	0.0041	45	3	5.80E-04	4.92E-03	NAPA,CLTC,DNM2
	Vibrio cholerae infection(K)	0.0047	51	3	8.32E-04	6.66E-03	ATP6V1H,ATP6V1C1,ATP6V1B1
	Signaling events mediated by VEGFR1 and VEGFR2(N)	0.0058	63	3	1.52E-03	0.0107	CTNNA1,PRKCB,DNM2
	Epithelial cell signaling in Helicobacter pylori infection(K)	0.0063	68	3	1.89E-03	0.0132	ATP6V1H,ATP6V1C1,ATP6V1B1
	Amphetamine addiction(K)	0.0063	68	3	1.89E-03	0.0132	CAMK2B,PRKCB,STX1A
	Adherens junction(K)	0.0066	72	3	2.22E-03	0.0134	PTPRF,CDH1,CTNNA1
	GABAergic synapse(K)	0.0081	88	3	3.90E-03	0.0208	NSF,GABBR1,PRKCB
	Rheumatoid arthritis(K)	0.0083	90	3	4.16E-03	0.0208	ATP6V1H,ATP6V1C1,ATP6V1B1
	Integration of energy metabolism(R)	0.0085	92	3	4.42E-03	0.0221	SNAP25,STXBP1,STX1A
	Retrograde endocannabinoid signaling(K)	0.0093	101	3	5.72E-03	0.0286	SLC17A6,SLC17A7,PRKCB
	Lysosome(K)	0.0113	123	3	9.78E-03	0.0403	CLTC,CLTB,ATP6V1H
	Oxidative phosphorylation(K)	0.0122	133	3	0.0121	0.0483	ATP6V1H,ATP6V1C1,ATP6V1B1
	Metabotropic glutamate receptor group III pathway(P)	0.0006	6	2	2.23E-04	3.34E-03	STX1B,STX1A
	Metabotropic glutamate receptor group II pathway(P)	0.0008	9	2	4.98E-04	4.58E-03	STX1B,STX1A
	LGI-ADAM interactions(R)	0.0013	14	2	1.19E-03	8.34E-03	STX1B,STX1A
	Arf1 pathway(N)	0.0018	20	2	2.40E-03	0.0144	CLTB,AP2M1
	E-cadherin signaling in keratinocytes(N)	0.0019	21	2	2.64E-03	0.0158	CDH1,CTNNA1
	Collecting duct acid secretion(K)	0.0025	27	2	4.30E-03	0.0215	ATP6V1C1,ATP6V1B1
	IL8- and CXCR1-mediated signaling events(N)	0.0026	28	2	4.62E-03	0.0231	PRKCB,DNM1
	Nectin adhesion pathway(N)	0.0028	30	2	5.28E-03	0.0264	CDH1,CTNNA1
	N-cadherin signaling events(N)	0.003	33	2	6.34E-03	0.0317	CTNNA1,CTTN
	Nicotine addiction(K)	0.0037	40	2	9.17E-03	0.0403	SLC17A6,SLC17A7
	TBC/RABGAPs(R)	0.0039	42	2	0.0101	0.0403	RAB11B,RAB7A
	Stabilization and expansion of the E-cadherin adherens junction(N)	0.0039	42	2	0.0101	0.0403	CDH1,CTNNA1
	Vasopressin-regulated water reabsorption(K)	0.004	44	2	0.011	0.044	NSF,RAB11B
	FGF signaling pathway(N)	0.0043	47	2	0.0125	0.0499	CDH1,CTTN
	AlphaE beta7 integrin cell surface interactions(N)	0.0003	3	1	0.0107	0.0428	CDH1

Supplementary Fig. 1.

Gene	UniProt ID (*H. sapiens*) / Aqu 2.1 model	Protein domain arrangement
ABL	P00519	SH3_1, SH2, Pkinase_Tyr, F_actin_bind
	Aqu2.1.31786_001	SH3_9, SH2, Pkinase_Tyr
AMPH	P49418	BAR, SH3_9
	Aqu2.1.37536_001	BAR, SH3_9
AP2	O94973	Adaptin_N, Alpha_adaptinC2, Alpha_adaptin_C
	Aqu2.1.08624_001	Adaptin_N, Alpha_adaptin..., Alpha_adaptin_C
	P63010	Adaptin_N, Alpha_adaptin..., B2-adapt-app_C
	Aqu2.1.39742_001	Adaptin_N, Alpha_a..., B2-adapt-app_C
	Q96CW1	Clat_adaptor_s, Adap_comp_sub
	Aqu2.1.18863_001	Clat_adaptor_s, Adap_comp_sub
	P53680	Clat_adaptor_s
	Aqu2.1.22337_001	Clat_adaptor_s
APBA	Q02410	PID, PDZ, PDZ
	Aqu2.1.23811_001	PID, PDZ
ATP2B2	Q01814	Cation_A..., E1-E2_ATPase, Hydrolase, Cation_ATPase_C, ATP_Ca...
	Aqu2.1.40972_001	Cation_A..., E1-E2_ATPase, Hydrolase, Cation_ATPase_C, AT...
ATP6V0	Q93050	V_ATPase_I
	Aqu2.1.24281_001	V_ATPase_I
	P27449	ATP-syn..., ATP-sy...
	Aqu2.1.25769_001	ATP-sy..., ATP-syn...
	Q99437	ATP-syn..., ATP-sy...
	Aqu2.1.33671_001	ATP-sy..., ATP-syn...
	P61421	vATP-synt_AC39
	Aqu2.1.38393_001	vATP-synt_AC39
	Q8NHE4	ATP_sy...
	Aqu2.1.40721_001	ATP_sy...
ATP6V1	P21283	V-ATPase_C
	Aqu2.1.20228_001	V-ATPase_C
	Q16864	ATP-synt_F
	Aqu2.1.22395_001	ATP-synt_F
	P15313	ATP-syn..., ATP-synt_ab, ATP-synt_a...
	Aqu2.1.26544_001	ATP-sy..., ATP-synt_ab, ATP-synt_ab_C
	Q9UI12	V-ATPase_H_N, V-ATPase_H_C
	Aqu2.1.27564_001	V-ATPase_H_N, V-ATPase_H_C
	Q9Y5K8	ATP-synt_D
	Aqu2.1.34794_001	ATP-synt_D
	P36543	vATP-synt_E
	Aqu2.1.41653_001	vATP-synt_E
	P38606	ATP-sy..., ATP-synt_ab, ATP-synt_ab_C
	Aqu2.1.41962_001	ATP-syn..., ATP-synt_ab, ATP-synt_a...

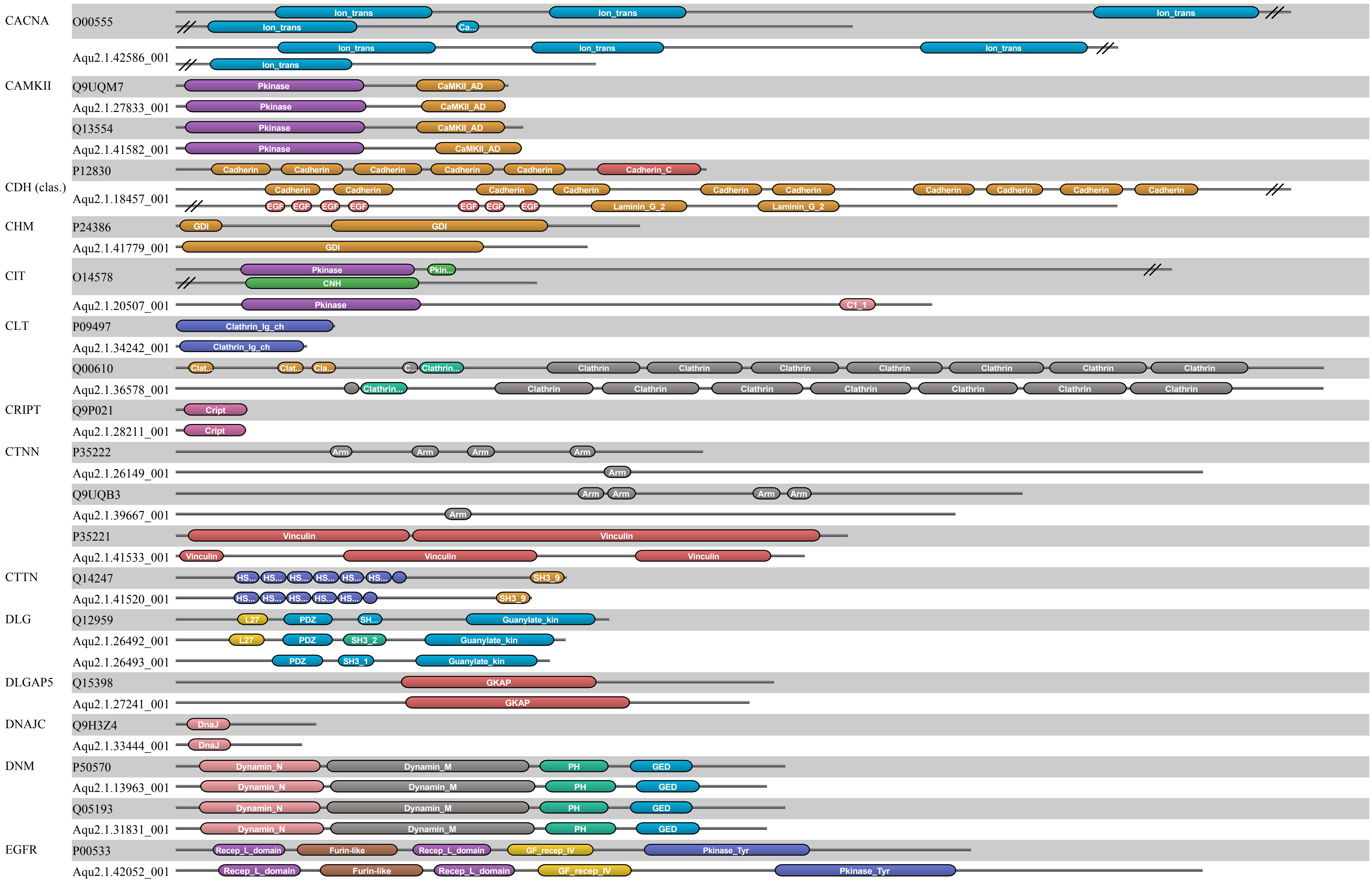
CACNA
O00555
Ion_trans
Ion_trans
Ion_trans
Ion_trans
Ca...
Aqu2.1.42586_001
Ion_trans
Ion_trans
Ion_trans
Ion_trans
CAMKII
Q9UQM7
Pkinase
CaMKII_AD
Aqu2.1.27833_001
Pkinase
CaMKII_AD
Q13554
Pkinase
CaMKII_AD
Aqu2.1.41582_001
Pkinase
CaMKII_AD
CDH (clas.)
P12830
Cadherin
Cadherin
Cadherin
Cadherin
Cadherin
Cadherin_C
Aqu2.1.18457_001
Cadherin
Cadherin
Cadherin
Cadherin
Cadherin
Cadherin
Cadherin
Cadherin
Cadherin
Cadherin
EGF
EGF
EGF
EGF
EGF
EGF
EGF
Laminin_G_2
Laminin_G_2
CHM
P24386
GDI
GDI
Aqu2.1.41779_001
GDI
CIT
O14578
Pkinase
Pkin...
CNH
Aqu2.1.20507_001
Pkinase
C1_1
CLT
P09497
Clathrin_lg_ch
Aqu2.1.34242_001
Clathrin_lg_ch
Q00610
Clat...
Clat...
Cla...
C...
Clathrin...
Clathrin
Clathrin
Clathrin
Clathrin
Clathrin
Clathrin
Clathrin
Aqu2.1.36578_001
Clathrin...
Clathrin
Clathrin
Clathrin
Clathrin
Clathrin
Clathrin
Clathrin
CRIPT
Q9P021
Cript
Aqu2.1.28211_001
Cript
CTNN
P35222
Arm
Arm
Arm
Arm
Aqu2.1.26149_001
Arm
Q9UQB3
Arm
Arm
Arm
Arm
Aqu2.1.39667_001
Arm
P35221
Vinculin
Vinculin
Aqu2.1.41533_001
Vinculin
Vinculin
Vinculin
CTTN
Q14247
HS...
HS...
HS...
HS...
HS...
HS...
SH3_9
Aqu2.1.41520_001
HS...
HS...
HS...
HS...
HS...
SH3_9
DLG
Q12959
L27
PDZ
SH...
Guanylate_kin
Aqu2.1.26492_001
L27
PDZ
SH3_2
Guanylate_kin
Aqu2.1.26493_001
PDZ
SH3_1
Guanylate_kin
DLGAP5
Q15398
GKAP
Aqu2.1.27241_001
GKAP
DNAJC
Q9H3Z4
DnaJ
Aqu2.1.33444_001
DnaJ
DNM
P50570
Dynamin_N
Dynamin_M
PH
GED
Aqu2.1.13963_001
Dynamin_N
Dynamin_M
PH
GED
Q05193
Dynamin_N
Dynamin_M
PH
GED
Aqu2.1.31831_001
Dynamin_N
Dynamin_M
PH
GED
EGFR
P00533
Recep_L_domain
Furin-like
Recep_L_domain
GF_recep_IV
Pkinase_Tyr
Aqu2.1.42052_001
Recep_L_domain
Furin-like
Recep_L_domain
GF_recep_IV
Pkinase_Tyr

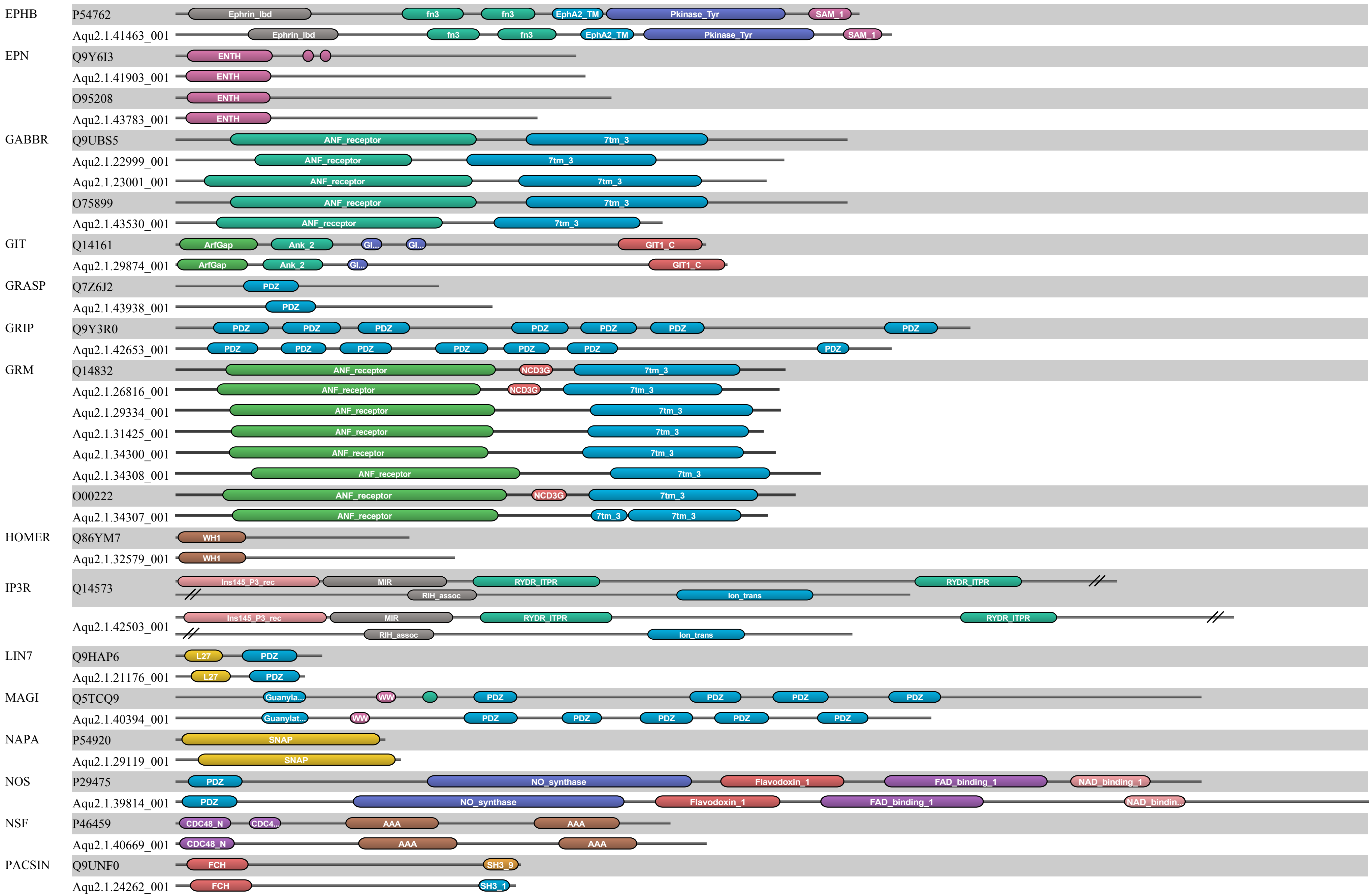

EPHB
P54762
Ephrin_lbd fn3 fn3 EphA2_TM Pkinase_Tyr SAM_1
Aqu2.1.41463_001
Ephrin_lbd fn3 fn3 EphA2_TM Pkinase_Tyr SAM_1
EPN
Q9Y6I3
ENTH
Aqu2.1.41903_001
ENTH
O95208
ENTH
Aqu2.1.43783_001
ENTH
GABBR
Q9UBS5
ANF_receptor 7tm_3
Aqu2.1.22999_001
ANF_receptor 7tm_3
Aqu2.1.23001_001
ANF_receptor 7tm_3
O75899
ANF_receptor 7tm_3
Aqu2.1.43530_001
ANF_receptor 7tm_3
GIT
Q14161
ArfGap Ank_2 GI... GI... GIT1_C
Aqu2.1.29874_001
ArfGap Ank_2 GI... GIT1_C
GRASP
Q7Z6J2
PDZ
Aqu2.1.43938_001
PDZ
GRIP
Q9Y3R0
PDZ PDZ PDZ PDZ PDZ PDZ PDZ
Aqu2.1.42653_001
PDZ PDZ PDZ PDZ PDZ PDZ PDZ
GRM
Q14832
ANF_receptor NCD3G 7tm_3
Aqu2.1.26816_001
ANF_receptor NCD3G 7tm_3
Aqu2.1.29334_001
ANF_receptor 7tm_3
Aqu2.1.31425_001
ANF_receptor 7tm_3
Aqu2.1.34300_001
ANF_receptor 7tm_3
Aqu2.1.34308_001
ANF_receptor 7tm_3
O00222
ANF_receptor NCD3G 7tm_3
Aqu2.1.34307_001
ANF_receptor 7tm_3 7tm_3
HOMER
Q86YM7
WH1
Aqu2.1.32579_001
WH1
IP3R
Q14573
Ins145_P3_rec MIR RYDR_ITPR RYDR_ITPR
RIH_assoc Ion_trans
Aqu2.1.42503_001
Ins145_P3_rec MIR RYDR_ITPR RYDR_ITPR
RIH_assoc Ion_trans
LIN7
Q9HAP6
L27 PDZ
Aqu2.1.21176_001
L27 PDZ
MAGI
Q5TCQ9
Guanyla... WW PDZ PDZ PDZ PDZ
Aqu2.1.40394_001
Guanylat... WW PDZ PDZ PDZ PDZ PDZ
NAPA
P54920
SNAP
Aqu2.1.29119_001
SNAP
NOS
P29475
PDZ NO_synthase Flavodoxin_1 FAD_binding_1 NAD_binding_1
Aqu2.1.39814_001
PDZ NO_synthase Flavodoxin_1 FAD_binding_1 NAD_bindin...
NSF
P46459
CDC48_N CDC4... AAA AAA
Aqu2.1.40669_001
CDC48_N AAA AAA
PACSIN
Q9UNF0
FCH SH3_9
Aqu2.1.24262_001
FCH SH3_1

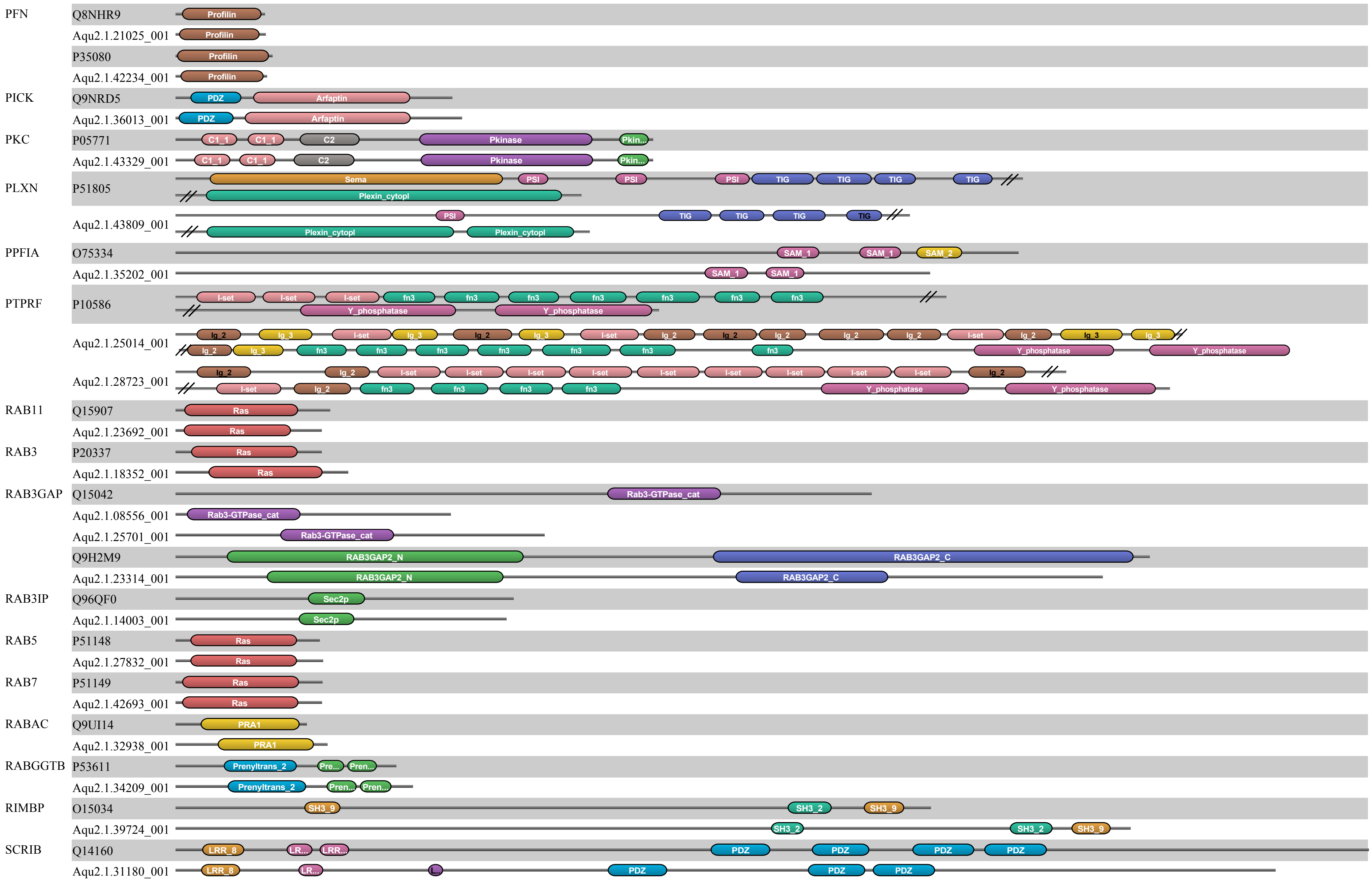

PFN
Q8NHR9 Profilin
Aqu2.1.21025_001 Profilin
P35080 Profilin
Aqu2.1.42234_001 Profilin
PICK
Q9NRD5 PDZ Arfaptin
Aqu2.1.36013_001 PDZ Arfaptin
PKC
P05771 C1_1 C1_1 C2 Pkinase Pkin...
Aqu2.1.43329_001 C1_1 C1_1 C2 Pkinase Pkin...
PLXN
P51805 Sema PSI PSI PSI TIG TIG TIG TIG Plexin_cytopl
Aqu2.1.43809_001 PSI TIG TIG TIG TIG Plexin_cytopl Plexin_cytopl
PPFIA
O75334 SAM_1 SAM_1 SAM_2
Aqu2.1.35202_001 SAM_1 SAM_1
PTPRF
P10586 I-set I-set I-set fn3 fn3 fn3 fn3 fn3 fn3 fn3 Y_phosphatase Y_phosphatase
Aqu2.1.25014_001 Ig_2 Ig_3 I-set Ig_3 Ig_2 Ig_3 I-set Ig_2 Ig_2 Ig_2 Ig_2 Ig_2 I-set Ig_2 Ig_3 Ig_3 Ig_2 Ig_3 fn3 fn3 fn3 fn3 fn3 fn3 fn3 Y_phosphatase Y_phosphatase
Aqu2.1.28723_001 Ig_2 Ig_2 I-set I-set I-set I-set I-set I-set I-set I-set I-set Ig_2 I-set Ig_2 fn3 fn3 fn3 fn3 Y_phosphatase Y_phosphatase
RAB11
Q15907 Ras
Aqu2.1.23692_001 Ras
RAB3
P20337 Ras
Aqu2.1.18352_001 Ras
RAB3GAP
Q15042 Rab3-GTPase_cat
Aqu2.1.08556_001 Rab3-GTPase_cat
Aqu2.1.25701_001 Rab3-GTPase_cat
Q9H2M9 RAB3GAP2_N RAB3GAP2_C
Aqu2.1.23314_001 RAB3GAP2_N RAB3GAP2_C
RAB3IP
Q96QF0 Sec2p
Aqu2.1.14003_001 Sec2p
RAB5
P51148 Ras
Aqu2.1.27832_001 Ras
RAB7
P51149 Ras
Aqu2.1.42693_001 Ras
RABAC
Q9UI14 PRA1
Aqu2.1.32938_001 PRA1
RABGGTB
P53611 Prenyltrans_2 Pre... Pren...
Aqu2.1.34209_001 Prenyltrans_2 Pren... Pren...
RIMBP
O15034 SH3_9 SH3_2 SH3_9
Aqu2.1.39724_001 SH3_2 SH3_2 SH3_9
SCRIB
Q14160 LRR_8 LR... LRR... PDZ PDZ PDZ PDZ
Aqu2.1.31180_001 LRR_8 LR... L... PDZ PDZ PDZ

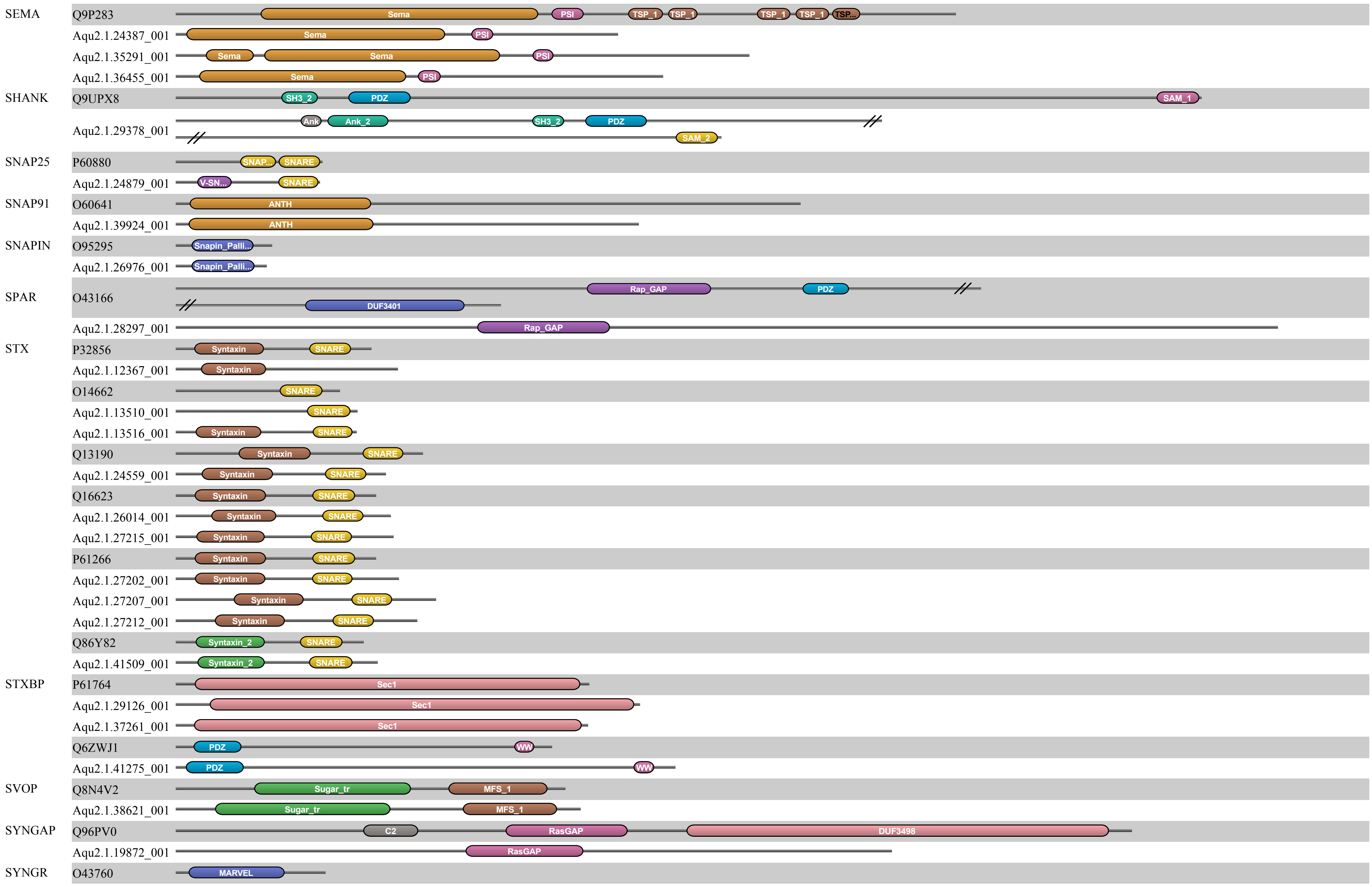
SEMA
Q9P283
Sema
PSI
TSP_1
TSP_1
TSP_1
TSP_1
TSP...
Aqu2.1.24387_001
Sema
PSI
Aqu2.1.35291_001
Sema
Sema
PSI
Aqu2.1.36455_001
Sema
PSI
SHANK
Q9UPX8
SH3_2
PDZ
SAM_1
Aqu2.1.29378_001
Ank
Ank_2
SH3_2
PDZ
SAM_2
SNAP25
P60880
SNAP...
SNARE
Aqu2.1.24879_001
V-SN...
SNARE
SNAP91
O60641
ANTH
Aqu2.1.39924_001
ANTH
SNAPIN
O95295
Snapin_Palli...
Aqu2.1.26976_001
Snapin_Palli...
SPAR
O43166
Rap_GAP
PDZ
DUF3401
Aqu2.1.28297_001
Rap_GAP
STX
P32856
Syntaxin
SNARE
Aqu2.1.12367_001
Syntaxin
O14662
SNARE
Aqu2.1.13510_001
SNARE
Aqu2.1.13516_001
Syntaxin
SNARE
Q13190
Syntaxin
SNARE
Aqu2.1.24559_001
Syntaxin
SNARE
Q16623
Syntaxin
SNARE
Aqu2.1.26014_001
Syntaxin
SNARE
Aqu2.1.27215_001
Syntaxin
SNARE
P61266
Syntaxin
SNARE
Aqu2.1.27202_001
Syntaxin
SNARE
Aqu2.1.27207_001
Syntaxin
SNARE
Aqu2.1.27212_001
Syntaxin
SNARE
Q86Y82
Syntaxin_2
SNARE
Aqu2.1.41509_001
Syntaxin_2
SNARE
STXBP
P61764
Sec1
Aqu2.1.29126_001
Sec1
Aqu2.1.37261_001
Sec1
Q6ZWJ1
PDZ
WW
Aqu2.1.41275_001
PDZ
WW
SVOP
Q8N4V2
Sugar_tr
MFS_1
Aqu2.1.38621_001
Sugar_tr
MFS_1
SYNGAP
Q96PV0
C2
RasGAP
DUF3498
Aqu2.1.19872_001
RasGAP
SYNGR
O43760
MARVEL

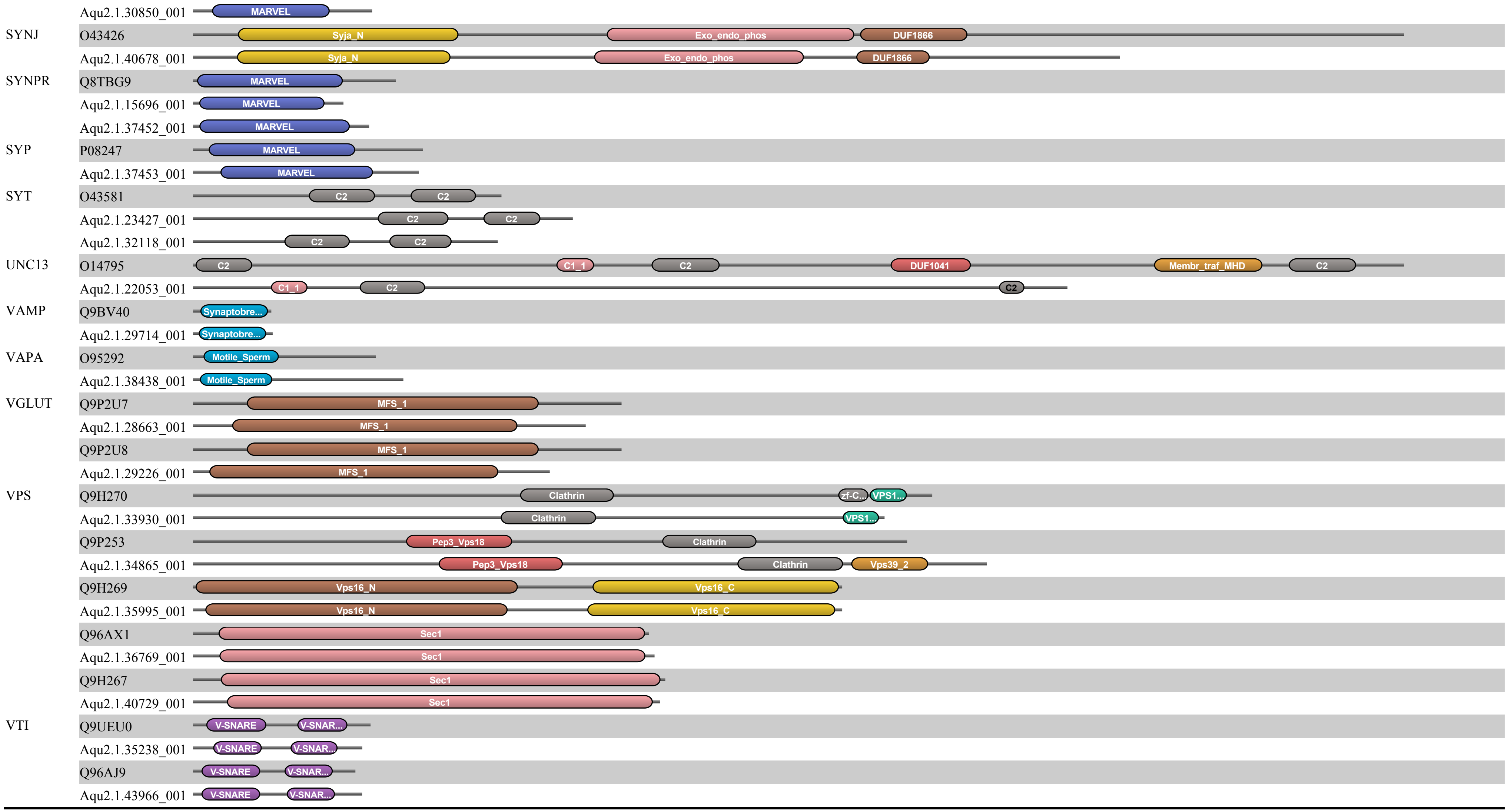

Aqu2.1.30850_001
MARVEL
SYNJ
O43426
Syja_N
Exo_endo_phos
DUF1866
Aqu2.1.40678_001
Syja_N
Exo_endo_phos
DUF1866
SYNPR
Q8TBG9
MARVEL
Aqu2.1.15696_001
MARVEL
Aqu2.1.37452_001
MARVEL
SYP
P08247
MARVEL
Aqu2.1.37453_001
MARVEL
SYT
O43581
C2
C2
Aqu2.1.23427_001
C2
C2
Aqu2.1.32118_001
C2
C2
UNC13
O14795
C2
C1_1
C2
DUF1041
Membr_traf_MHD
C2
Aqu2.1.22053_001
C1_1
C2
C2
VAMP
Q9BV40
Synaptobre...
Aqu2.1.29714_001
Synaptobre...
VAPA
O95292
Motile_Sperm
Aqu2.1.38438_001
Motile_Sperm
VGLUT
Q9P2U7
MFS_1
Aqu2.1.28663_001
MFS_1
Q9P2U8
MFS_1
Aqu2.1.29226_001
MFS_1
VPS
Q9H270
Clathrin
zf-C...
VPS1...
Aqu2.1.33930_001
Clathrin
VPS1...
Q9P253
Pep3_Vps18
Clathrin
Aqu2.1.34865_001
Pep3_Vps18
Clathrin
Vps39_2
Q9H269
Vps16_N
Vps16_C
Aqu2.1.35995_001
Vps16_N
Vps16_C
Q96AX1
Sec1
Aqu2.1.36769_001
Sec1
Q9H267
Sec1
Aqu2.1.40729_001
Sec1
VTI
Q9UEU0
V-SNARE
V-SNAR...
Aqu2.1.35238_001
V-SNARE
V-SNAR...
Q96AJ9
V-SNARE
V-SNAR...
Aqu2.1.43966_001
V-SNARE
V-SNAR...

Supplementary Fig. 2.

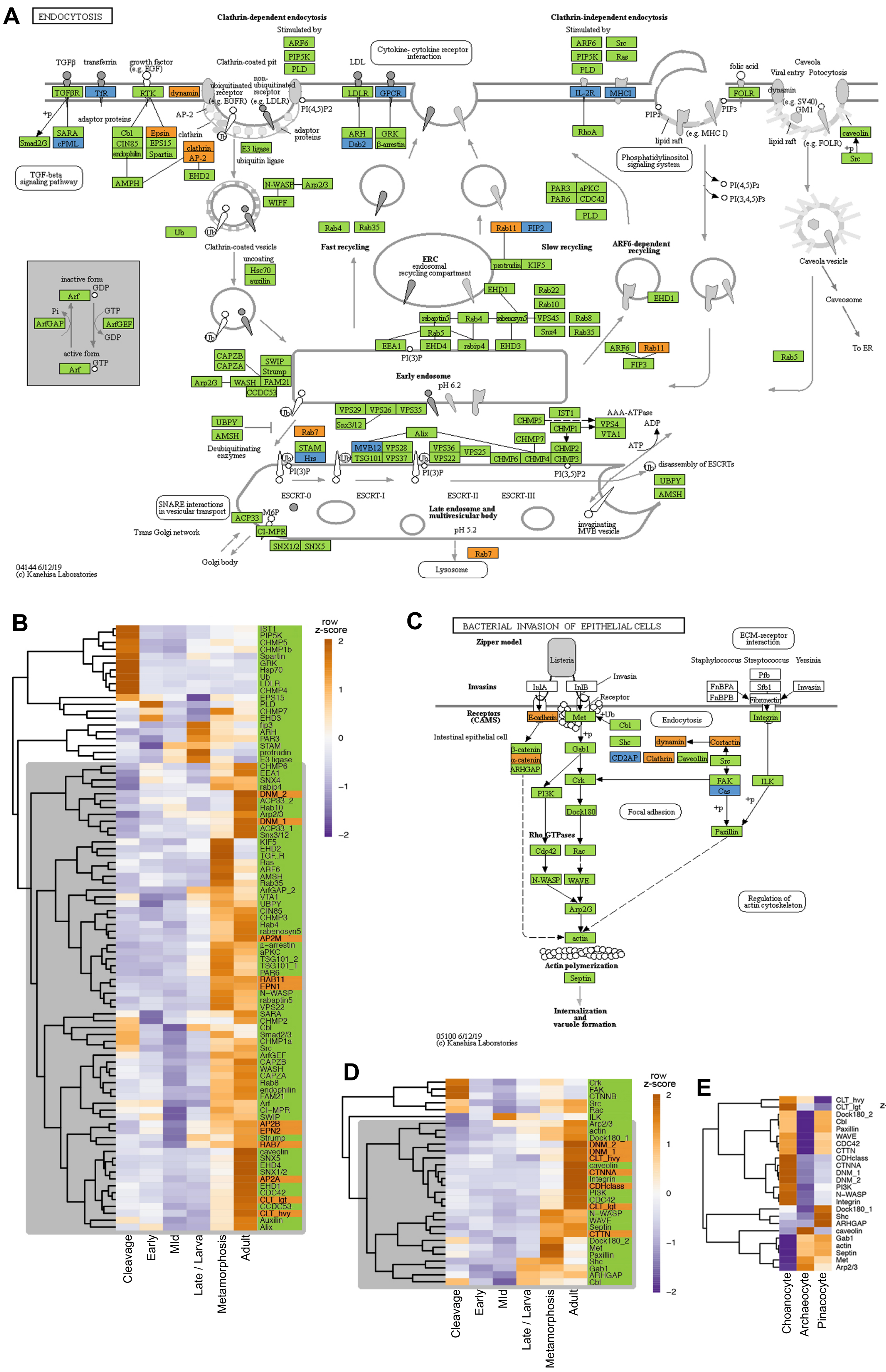

Supplementary Fig. 3.

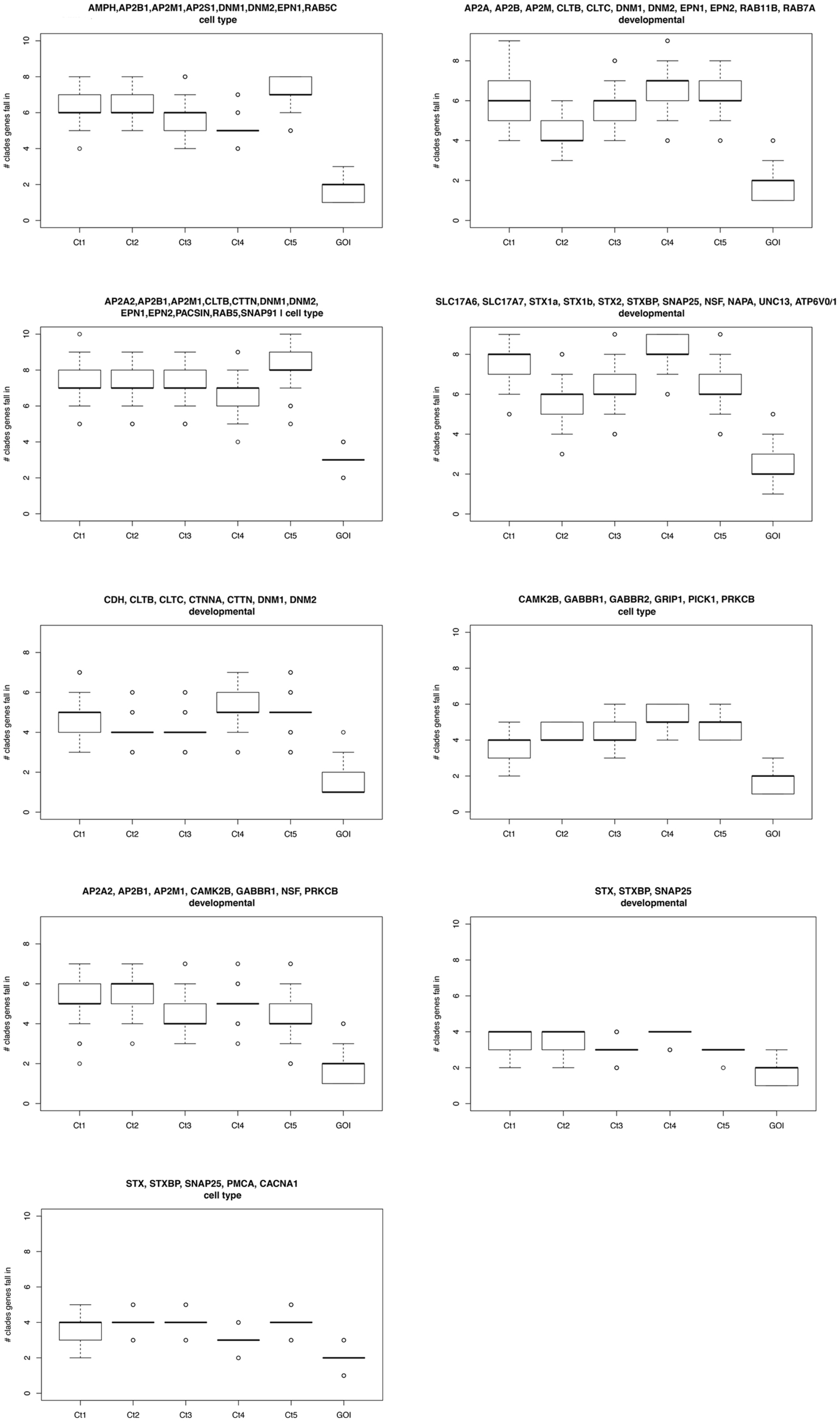

Supplement: Supplementary file 1 — Supplementary Information Total [file 41598_2019_51282_MOESM1_ESM.pdf]
